# Targeted protein degradation by Trim-Away using cell resealing coupled with microscopic image-based quantitative analysis

**DOI:** 10.3389/fcell.2022.1027043

**Published:** 2022-12-19

**Authors:** Rina Kunishige, Masayuki Murata, Fumi Kano

**Affiliations:** ^1^ Cell Biology Center, Institute of Innovative Research, Tokyo Institute of Technology, Yokohama, Japan; ^2^ Multimodal Cell Analysis Collaborative Research Cluster, Tokyo Institute of Technology, Yokohama, Japan; ^3^ International Research Center for Neurointelligence, Institutes for Advanced Study, The University of Tokyo, Tokyo, Japan

**Keywords:** protein degradation, protein targeting, antibody, cell permeabilization, microscopic imaging, Trim-Away, cell-resealing technique

## Abstract

“Trim-Away” technology enables rapid degradation of endogenous proteins without prior modification of protein-coding genes or mRNAs through delivery of antibodies that target proteins of interest. Although this approach can be readily applied to almost any cytosolic protein, strategies for cytosolic antibody delivery have been limited to microinjection or electroporation, which require skill-dependent operation or specialized equipment. Thus, the development of antibody delivery methods that are convenient, scalable, and preferably do not require detachment of adherent cells is required to extend the versatility of the Trim-Away method. Here, we developed a cell resealing technique optimized for Trim-Away degradation, which uses the pore-forming toxin streptolysin O (SLO) to permeabilize the cell membrane and delivered the antibodies of interest into HEK293T, HeLa, and HK-2 cell lines. We demonstrated the ability of Trim-Away protein degradation using IKKα and mTOR as targets, and we showed the availability of the developed system in antibody screening for the Trim-Away method. Furthermore, we effectively coupled Trim-Away with cyclic immunofluorescence and microscopic image-based analysis, which enables single-cell multiplexed imaging analysis. Taking advantage of this new analysis strategy, we were able to compensate for low signal-to-noise due to cell-to-cell variation, which occurs in the Trim-Away method because of the heterogenous contents of the introduced antibody, target protein, and TRIM21 in individual cells. Therefore, the reported cell resealing technique coupled with microscopic image analysis enables Trim-Away users to elucidate target protein function and the effects of target protein degradation on various cellular functions in a more quantitative and precise manner.

## Introduction

Intracellular delivery of antibodies has become a powerful tool for studying endogenous protein function. For example, fluorescent-labeled antibodies can be used to visualize endogenous target proteins in live cells (Teng et al., 2016). Additionally, antibodies can be used to inhibit the functions of proteins by binding to their targets (Sonoda et al., 2021). Clift et al. (2017), Clift et al. (2018) reported another approach where the intrinsic function of TRIM21, a ubiquitin ligase that binds to the Fc domain of antibodies, was used to target protein degradation *via* the proteasome. This “Trim-Away” method enables rapid protein degradation with a half-life of 10–20 min and without prior modification of the target protein (Clift et al., 2017, Clift et al., 2018). The Trim-Away process consists of the following three steps: (1) the antibody against the target protein is introduced into the cytosol, (2) TRIM21 is recruited to the antibody-bound target protein, and (3) the TRIM21–antibody–target complex is degraded by the proteasome (Clift et al., 2017, 2018). Compared with other mainstream targeted protein degradation modalities such as proteolysis-targeting chimeras (PROTACs) (Sakamoto et al., 2001), there is no need to design a degrader molecule for each protein of interest for Trim-Away in most cases because commercially available off-the-shelf antibodies can be used to target a wide range of proteins. In addition to the versatility of Trim-Away, the speed of the method enables the analysis of the direct consequences of protein knockdown before any compensatory mechanism is activated (Clift et al., 2017, 2018).

Previously, cytosolic antibody delivery has been achieved mainly *via* capillary electroporation and microinjection (Clift et al., 2017, 2018; Masumoto et al., 2017; Chen et al., 2019; Kiss et al., 2019; Mehlmann et al., 2019; Zhou et al., 2019; Eisa et al., 2020; Yang et al., 2020; Weir et al., 2021). However, these approaches have low throughput and require special skills and/or equipment; thus, they may have hindered the extensive use of Trim-Away. Further, capillary electroporation requires the cells to be suspended, which hinders rapid observations or assays because adherent cells require ∼4 h to completely re-adhere (Clift et al., 2017). Therefore, in the present study, we developed and tested a new approach for antibody delivery where cells are kept adherent throughout the procedure. To achieve this, we used a cell resealing technique: a simple and high-throughput technique that does not require special skills or equipment to perform (Kano and Murata, 2013; Kunishige et al., 2020).

To date, cell resealing has been used for the intracellular delivery of various membrane-impermeable molecules, such as chemical compounds, nucleotides, peptides, and soluble proteins (Murakami et al., 2018; Yamaoki et al., 2018; Sonoda et al., 2021). The technique involves first treating the cell membrane with a cholesterol-dependent pore-forming toxin, such as treatment with streptolysin O (SLO) at 4°C. In this example, when the temperature is increased to 32°C–37°C, the membrane-bound SLO is oligomerized and forms a ring-shaped multimer, creating approximately 30-nm-diameter pores in the plasma membrane. Such membrane-permeabilized cells are referred to as “semi-intact cells,” which allow the introduction of various molecules with a range of molecular sizes, including IgG (∼150 kDa), other proteins, or fluorescent-labeled dextran, into the cells. In addition, the pores can be rapidly resealed within a few minutes through incubation with cytosol and calcium ions. Herein, we refer to these cells as “resealed cells” and the procedure used to produce such cells as the “cell resealing technique.” This technique is scalable because only the volume of the buffers needs to be adjusted to conduct experiments on a small (e.g., in 384-well plate) or large (larger culture dishes) scale, and this allows for the analysis of 1–10^7^ cells, morphologically and biochemically, *via* various microscopy and omics methods, respectively.

Although cell resealing is a robust and widely applicable method, the need for supplementing exogenous cytosol obtained from a donor cell line, which is often different from the host cells, is a drawback of this technique. This is not only because of the burden of obtaining cytosol but also because the physiology of the host cell might be perturbed by the inflow of donor cytosol. Especially when using knockout or knockdown cells for host cells, the particular proteins whose expression was inhibited may flow in from the exogenous cytosol, complicating the interpretation of the results. Here, we aimed to overcome this complication by establishing a simplified resealing protocol that minimizes the inflow of exogenous cytosol. We also aimed to develop a more convenient, small-scale cytosol preparation method, which enabled us to obtain cytosol from the same cell line as the host cell. Another complication of cell resealing is the presence of cell-to-cell variation in the permeabilization/resealing efficiency, which is likely derived from variation in the cholesterol content of the plasma membrane. Such variations can lower the signal-to-noise ratio of the analysis because the combined results reflect both efficiently and inefficiently resealed cells. In these cases, immunofluorescence and single-cell analysis can be effectively applied to handle the cell-to-cell variation (Kano et al., 2017; Kunishige et al., 2020; Noguchi et al., 2021). For example, fluorescently labeled dextran has been used as a marker for permeabilization efficiency; this helps eliminate data on non-permeabilized cells and thereby improves the signal-to-noise ratio (Kano et al., 2017). Thus, the combination of immunofluorescence and single-cell analysis is an effective approach for analyzing resealed cells because it enables data collection on, for example, abundance (quantity), post-translational modification state (quality), and protein localization. Such data can be used to gain further insights, particularly when the correlations among the feature data are determined.

In the Trim-Away method, in addition to the cell-to-cell variation of the introduced antibodies, variation in target and TRIM21 protein amounts affects the efficiency of Trim-Away protein degradation. However, single-cell immunofluorescence analysis provides a means to deal with this cell-to-cell variation. Additionally, the cyclic immunofluorescence method enables multiplexed imaging *via* a procedure involving repeated staining and bleaching; this should be an effective method when coupled with Trim-Away (Lin et al., 2015, 2016; Radtke et al., 2020, 2022). In the present study, we established a widely applicable cell resealing protocol to deliver antibodies for Trim-Away-mediated protein degradation. By combining Trim-Away with cyclic immunofluorescence and microscopic image-based quantitative analysis, we developed a rapid, precise, and scalable Trim-Away analysis system.

## Materials and methods

### Cell culture and reagents

The TRIM21-overexpressed Piranha^TM^ HEK293T cells GFP/Puromycin Stable Cell Line (#PTPD600A-1) was purchased from System Biosciences (CA, United States). We cultured HEK293T cells and Piranha^TM^ HEK293T cells in Dulbecco’s modified essential medium (DMEM) high-glucose medium (#041-30081; Fujifilm) supplemented with 10% fetal bovine serum (FBS), 2 mM of GlutaMax (#35050-079; Gibco, Grand Island, NY, United States), and 100 U/mL of penicillin and 100 μg/ml of streptomycin (#15140-122; Gibco) in 5% CO_2_ at 37°C. We cultured HeLa cells in DMEM (#05915; Nissui Pharmaceutical, Tokyo, Japan) supplemented with 10% FBS and 100 U/mL of penicillin and 100 μg/ml of streptomycin in 5% CO_2_ at 37°C. HeLa-GFP-LC3 cells and HeLa-GFP-CD63 cells, which continuously express GFP-LC3 and GFP-CD63, respectively, were grown in the same medium with the addition of 500 μg/ml of Geneticin (#11811023; Gibco). The human renal proximal tubular epithelial cell line HK-2 (ATCC CRL-2190) was purchased from the American Type Culture Collection (Manassas, VA, United States). We cultured HK-2 cells in DMEM and Ham’s F-12 medium (DMEM/F-12) (#11320082; Gibco) containing 10% FBS and 100 U/ml of penicillin and 100 μg/ml of streptomycin (#15140-122; Gibco) in 5% CO_2_ at 37°C. For details of the reagents used in this study, see the Supporting Information.

### TRIM21 protein purification

TRIM21 protein was purified according to methods described previously with slight modification (Clift et al., 2018). *E. coli* C41(DE3) cells were transformed with HLTV-hTRIM21 plasmids (Addgene, plasmid no. 104973), and His-Lipoyl-TRIM21 protein expression was induced with 0.1 mM IPTG overnight at 22°C. Cell pellets were sonicated in lysis buffer (300 mM KCl, 50 mM KH_2_PO_4_, 5 mM imidazole, pH 8.0) supplemented with complete protease inhibitors (Roche), followed by the addition of 0.5% Triton X-100 and rotation for 30 min. Following 15,000 rpm centrifugation for 20 min, the supernatant was filtered through a 0.22 µm filter, and His-Lipoyl-TRIM21 protein was purified using a 5 ml Bio-Scale Mini Profinia IMAC cartridge (Bio-rad) and the Profinia Protein Purification System according to standard Bio-rad protocols. Protein was further purified in a HiLoad 16/600 Superdex 200 PG size exclusion column using an AKTA pure purification system (GE Healthcare). Peak fractions were pooled and concentrated using Amicon Ultra-4 50 kDa (Millipore), and the buffer was exchanged to PBS. Proteins were analyzed by 5%–20% gradient sodium dodecyl sulfate polyacrylamide gel electrophoresis (SDS–PAGE) and Coomassie Brilliant Blue staining. His-Lipoyl-TRIM21 protein aliquots were frozen and stored at −80°C.

### Preparation of cytosol

We prepared cytosol from murine lymphoma L5178Y cells as described previously (Kano et al., 2012).

Cytosol from HEK293T cells, HeLa cells, and HK-2 cells was obtained by freeze-thawing. First, HeLa cells or HEK293T cells grown on a 10 cm dish were trypsinized and resuspended in complete medium. The cells were washed twice with transport buffer (TB) [25 mM 4-(2-hydroxyethyl)-1-piperazineethanesulfonic acid-potassium hydroxide (pH 7.4), 115 mM potassium acetate, 2.5 mM MgCl_2_, and 2 mM ethylene glycol-bis(β-aminoethyl ether)-N,N,N,N-tetra-acetic acid], and then resuspended in 100 μl TB. Following three freeze-thaw cycles and 12,000 rpm centrifugation for 10 min, the supernatant was collected as the cytosol and stored at −80°C.

### Cell resealing

The antibodies used for resealing were ultrafiltered to remove cytotoxic preservatives, such as sodium azide, as described previously (Clift et al., 2018). We developed the cell resealing protocol optimized for intracellular antibody introduction by modifying the resealing protocol described previously (Kano et al., 2012, 2017). HeLa cells and HK-2 cells were grown on 96-well dishes (#655090; Greiner Bio-One), and HEK293T cells and Piranha^TM^ HEK293T cells were grown on poly-D-lysine-coated 96-well dishes (#354640; Corning). The cells were washed with PBS, after which they were incubated with ∼0.4 μg/ml of SLO (#01-531; Bioacademia, Osaka, Japan) for 5 min on ice. Note that the SLO concentration should be routinely optimized because differences in cell number, nutrition conditions, or lot-to-lot variation in SLO activity affect permeabilization efficiency. After another wash, the cells were incubated with TB for 5 min at 37°C. Membrane-permeabilized cells were washed once with TB, after which they were incubated with the antibodies of interest and 3.0–6.0 mg/ml of cytosol supplemented with an ATP-regenerating system (1 mM ATP, 50 µM creatine kinase, and 2.62 mg/ml creatine phosphate), 1 mg/ml of glucose, and 1 mM GTP for 10 min at 37°C. For resealing, the cells were incubated with 1 mM CaCl_2_ and 3.0–6.0 mg/ml of cytosol supplemented with an ATP-regenerating system for 5 min at 37°C. The resealed cells were further incubated with growth medium in a 5% CO_2_ incubator at 37°C for the time indicated in the figure legends. Note that although the supplementation of cytosol is preferred for the resealing step for higher retention rate of the introduced antibody, cytosol in the antibody introduction step is optional, as the absence of cytosol does not affect resealing efficiency.

Two-step resealing was performed based on the protocol described above, except for the additional TRIM21 protein introduction step. Briefly, cells were treated with ∼0.4 μg/ml of SLO on ice for 5 min and incubated with prewarmed TB at 37°C for 5 min. Then, the semi-intact cells were incubated with antibody supplemented with an ATP-regenerating system for 10 min, followed by incubation with 6.5 mg/ml TRIM21 protein supplemented with an ATP-regenerating system for 5 min. Cells were then resealed by incubation with 1 mM CaCl_2_ and 3.0–6.0 mg/ml of cytosol supplemented with an ATP-regenerating system for 5 min at 37°C and allowed to recover in the incubator at 37°C.

For resealing experiments using fluorophore-conjugated antibodies, the cells were imaged with a ×20 or ×40 objective using Cytation 5 reader and Gen5 software. Whole-well images were obtained by stitching the images.

For resealing experiments using a proteasome inhibitor, the cells were pretreated with 25 µM MG132 (Fujifilm; #139-18451) for 1–3 h and subjected to resealing procedures. MG132 was supplemented during the resealing steps and during incubation after resealing.

### Adherent electroporation

Piranha^TM^ HEK293T cells grown on poly-D-lysine-coated 96-well dishes (#354640; Corning) or HeLa cells grown on 96-well dishes (#655090; Greiner Bio-One) were electroporated using a Super Electroporator NEPA21 Type II (Nepagene, Chiba, Japan) and cell-culture-plate electrode (#CUY900-5-2-3; Nepagene) with 2 mm between electrodes. Cells were first washed with Opti-MEM, after which they were electroporated in 50 μl of PBS containing the antibodies of interest. The parameters for electroporation were as follows: the polarity exchanged poring pulse: 125–225 V, 2.5 m, two pulses with intervals of 50 m, and a 10% attenuation rate; the following polarity exchanged transfer pulse: 30 V, 50 m, five pulses with intervals of 50 m, and a 40% attenuation rate. This electroporation program was performed twice, and the electrode was rotated 90° the second time. Cells were incubated with growth medium in a 5% CO_2_ incubator at 37°C for the time indicated in the figure legends.

### Cuvette electroporation

HeLa cells grown on 6-cm dishes were washed with Opti-MEM, and 1 × 10^5^ cells were resuspended in 20 μL of PBS containing the antibodies of interest. The cells were then transferred to a cuvette with a 2-mm gap (#EC-002S; Nepagene) and electroporated using the following settings: poring pulse: 50–150 V, 2.5 m, two pulses with intervals of 50 m, and a 10% attenuation rate; the following polarity exchanged transfer pulse: 20 V, 50 m, five pulses with intervals of 50 m, and a 40% attenuation rate. After 600 μl of growth medium was added to the cuvette, 100 μL of the cell suspension was seeded in a 96-well dish. Cells were incubated in a 5% CO_2_ incubator at 37°C for the time indicated in the figure legend.

### Small interfering RNA and transfection

siRNA against human IKKα (#SASI_Hs01_00206,922), siRNA against human TRIM21 (#SASI_Hs01_00060144) were purchased from Sigma Aldrich. Negative control siRNA (Silencer Negative Control 1 siRNA; #AM4635) was obtained from Ambion, Piranha HEK 293T cells were transfected using Lipofectamine^TM^ RNAiMAX (Invitrogen, CA, United States) according to the manufacturer’s instructions.

### Western blotting assay

HEK293T, HeLa, and HK-2 cells grown in 96-well dishes were lysed with 40 μL of ice-cold radioimmunoprecipitation assay buffer [1% Triton X-100, 0.1% sodium dodecyl sulfate, 1% sodium deoxycholate, 150 mM NaCl, 50 mM Tris-HCl (pH 8.0)] supplemented with 1 mM PMSF. After the addition of 40 μl 2× Laemmli’s sodium dodecyl sulfate sample buffer, the lysates were boiled for 5 min and subjected to 5%–20% gradient sodium dodecyl sulfate polyacrylamide gel electrophoresis. Following electrophoresis, proteins were transferred to a PVDF membrane using a Criterion blotter (Bio-Rad) at 70 V for 120–180 min, after which the membrane was blocked with 5% (w/v) bovine serum albumin. The membrane was probed with primary antibodies at 4°C overnight and incubated with horseradish peroxidase-conjugated secondary antibodies at room temperature for 1 h. Finally, the proteins were detected with a Western Lightning Plus-ECL system (PerkinElmer) using LAS4000 mini (Fujifilm).

### Immunofluorescence, cyclic immunofluorescence, and microscopy

HEK293T, HeLa, and HK-2 cells grown in 96-well dishes were fixed in 4% paraformaldehyde for 15 min, after which they were permeabilized using 0.2% Triton X-100 in PBS for 5 min at room temperature. After the cells were blocked with 3% bovine serum albumin for 30 min, they were incubated with primary antibodies in blocking buffer for >2 h, washed three times with PBS, and further incubated with fluorophore-conjugated secondary antibodies and Hoechst 33342 in blocking buffer for 1 h at room temperature. The cells were washed three times with PBS, and images were obtained using a ×40 objective and a Nikon A1 confocal laser scanning microscope (Nikon, Tokyo, Japan). During automated image acquisition using NIS-Elements software, Hoechst 33342 was used for autofocusing.

After the first round of imaging, the fluorophores of the secondary antibodies were bleached using a mixture of 4.5% H_2_O_2_ and 25 mM NaOH in PBS for 1 h at room temperature with exposure to light from a table lamp (Lin et al., 2015, 2016). The cells were then washed three times with PBS, and a second round of immunofluorescence was performed using fluorescently labeled primary antibodies. The labeled antibodies were prepared using a Zenon Labeling Kit according to the manufacturer’s instructions (Invitrogen, CA, United States). Labeled antibodies were diluted with blocking buffer and used immediately. After the cells had been incubated with fluorescently labeled primary antibodies for 1 h, they were once again imaged with the Nikon A1 confocal laser scanning microscope. The same position in the well was imaged for all rounds of cyclic immunofluorescence, and the Hoechst images were used as references to generate registration.

After the second round of imaging, the fluorophores of the Zenon-labeled antibodies were bleached using a mixture of 3% H_2_O_2_ and 20 mM NaOH in PBS for 1 h at room temperature with exposure to light from a table lamp. The same procedure was performed after the third cycle of immunofluorescence.

### Image analysis and visualization

We analyzed the microscopic images using NIS-Elements software Ver5.11 (Nikon, RRID:SCR_014329), performing maximum intensity projection for z-stack images. Cyclic immunofluorescence images acquired from different rounds of imaging were aligned and combined using the Hoechst images from every round as references.

Single-cell analysis was performed by first detecting the areas of the nucleus using binary images in the Hoechst channel and then detecting the cell area by the thickening of the nucleus area. We measured the mean or sum fluorescence intensity of the cell area for all channels. Intensity data were passed to R version 4.0.5 with RStudio (version 2021.09.1 + 372; RStudio Inc., MA, United States) for further analysis and visualization. The R packages “ggplot2” version 3.3.5 and “GGally” version 2.1.2 were used to generate pairwise plots. The dimensionality reduction algorithm t-SNE was implemented using “Rtsne” version 0.15.

For colocalization analysis, segmentation of the cytosol region was performed using NIS-Elements software, and Pearson’s correlation coefficient between the indicated channels was calculated using the pixel intensity of the cytosol region for each cell.

### Statistical analysis

We assessed the statistical significance of differences from the control using Dunnett’s test as a multiple comparison test, and the differences between the two groups were detected using Welch’s t-test. In Figure 3C, we assessed the differences between individual sets of data using Tukey’s tests. *p* < 0.05 was considered statistically significant. Results are reported as means ± standard errors of the mean, and the number of samples used and analyzed is shown in each figure legend.

## Results

### Application of the cell resealing technique-mediated antibody delivery for Trim-Away

**FIGURE 1:**
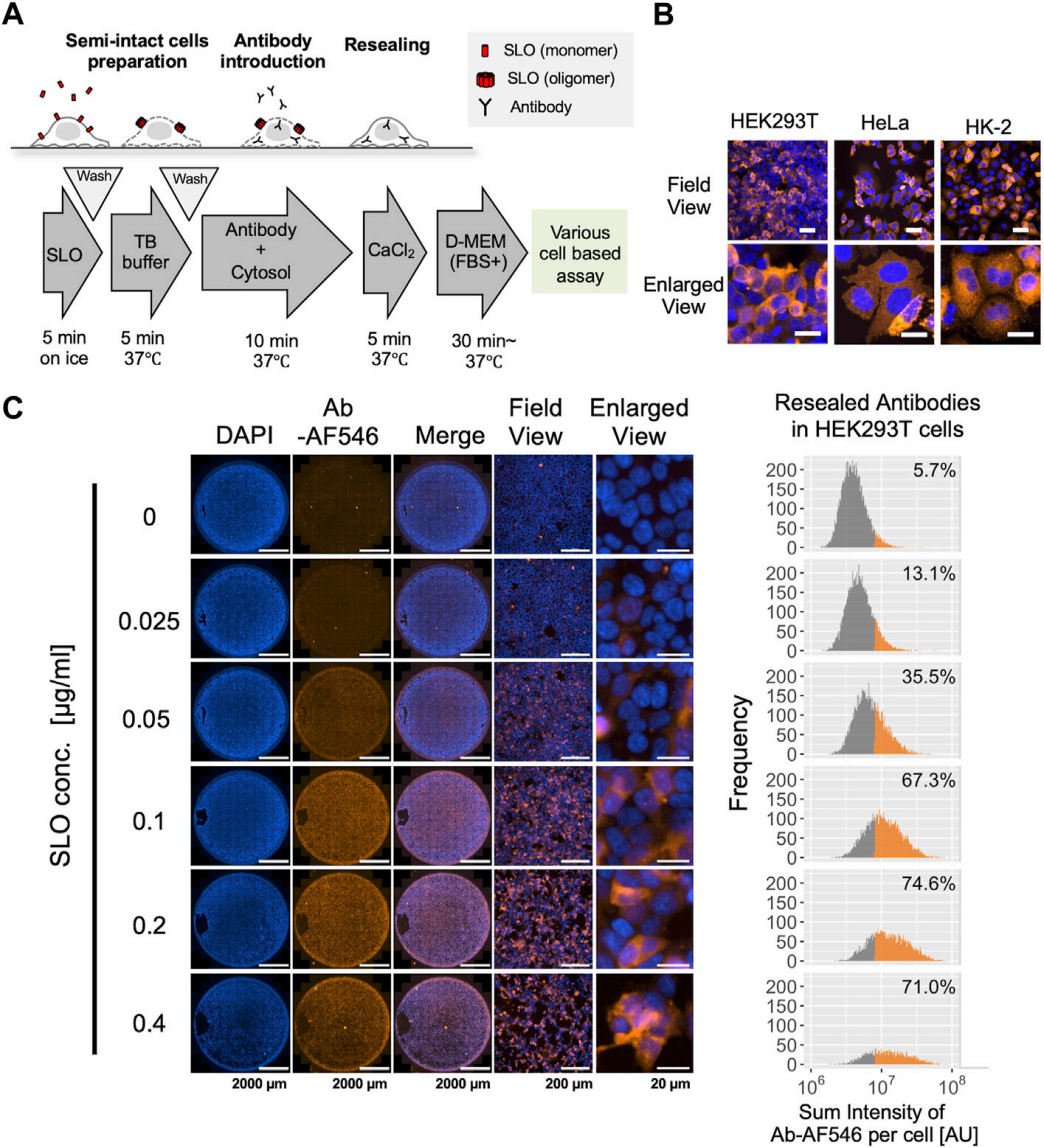
Cytosolic delivery of antibodies using the cell resealing technique. **(A)** Outline of the streptolysin O (SLO)-mediated cell resealing technique. Cells were treated with ∼0.4 μg/ml of SLO on ice for 5 min and incubated with prewarmed transport buffer (TB) at 37°C for 5 min, and the permeabilized cells were incubated with the antibody and exogenous cytosol supplemented with an ATP-regenerating system, GTP, and glucose for 10 min. Cells were then resealed by incubation with 1 mM CaCl_2_ for 5 min at 37°C and allowed to recover in the incubator at 37°C. **(B)** Confocal microscopy image of antibody-resealed HEK293T, HeLa, and HK-2 cells, fixed 3 h after resealing and stained with secondary antibody. Antibodies were successfully introduced without excessive cell damage. Scale bars: 50 μm (top panel); 20 μm (bottom panel). **(C)** Evaluation of optimal SLO concentration in HEK293T cells. Cells were permeabilized with 0.025, 0.05, 0.1, 0.2, or 0.4 μg/ml of SLO, and Alexa Fluor 546-labeled non-targeting antibody (Ab-AF546) was resealed according to the protocol outlined in A. Cells were fixed 1 h later and imaged using Cytation 5. Whole-well images show that the labeled antibodies were introduced throughout the well from the center to the periphery. Histograms (right) show the sum fluorescence intensities of the antibodies measured per cell and the percentage of fluorescently labeled antibody-positive cells. Low and high SLO concentrations resulted in inefficient permeabilization and excessive cell damage, respectively, leading to a decrease in live cells. The optimal SLO concentration was 0.1 μg/ml for HEK293T cells. Ab: antibody, AU: arbitrary unit. Scale bar: 2,000 μm (Well view); 200 μm (Field view); 20 μm (Enlarged view).

In our previous studies, we attempted to apply semi-intact cells or a cell resealing technique to deliver antibodies and inhibit their intracellular targets (Kano et al., 2000; Sonoda et al., 2021). However, the standard protocols used in these studies were not thoroughly optimized for antibody delivery, and the actual efficacy or retention of the delivered antibodies has not yet been explored in detail. Therefore, we first established a cell resealing protocol for antibody delivery using three cell lines: HEK293T, HeLa, and HK-2 (Figure 1A). As shown in Figure 1B, the antibodies were successfully introduced without excessive cell damage in all three cell lines. In these resealing experiments, the optimal SLO concentration was first determined for each of the three cell lines using single-cell analysis and microscopic images (Figure 1C, Supplementary Figure S1). For HEK293T cells, 0.2 μg/ml was the optimal SLO concentration, which successfully delivered antibodies in 74.6% of the cells (Figure 1C). We also confirmed that the delivered antibody could successfully bind to the intracellular target by resealing the anti-GFP antibody in HeLa cells expressing CD63-GFP or LC3-GFP (Supplementary Figure S2). Next, we tested the retention of the resealed antibodies for up to 24 h, confirming that almost half of the antibodies were retained in the cells at 24 h after resealing (Supplementary Figure S3A). The membrane resealing efficiency was determined for the three cell lines using the membrane-impermeable dye propidium iodide as an indicator. The results indicated that most cells efficiently resealed the pores immediately after the resealing step, whereas few cells (∼1%) exhibited incomplete resealing (Supplementary Figure S4). We also observed the resealed cells up to 48 h and confirmed that these cells exhibited normal shapes and survived for at least 48 h (Supplementary Figure S5). Taking advantage of the ability of this cell resealing technique to simultaneously introduce a variety of antibodies, we introduced three different antibodies (IgGs) into HEK293T cells using a single resealing procedure (Supplementary Figure S3B).

**FIGURE 2:**
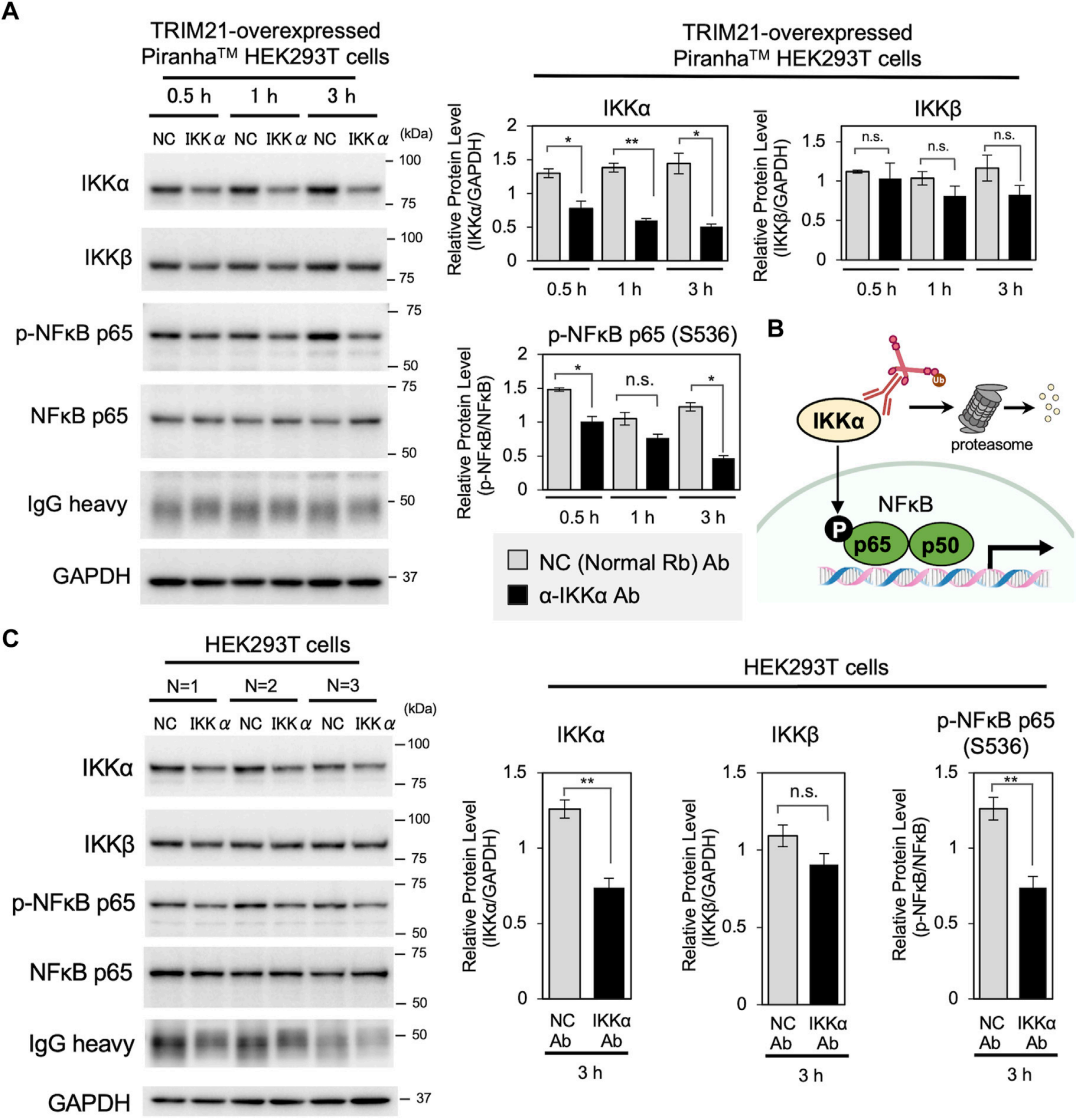
Acute degradation of IKKα by cell resealing technique-mediated Trim-Away. **(A)** Western blotting showing acute degradation of IKKα by cell resealing technique-mediated Trim-Away. TRIM21-overexpressed Piranha^TM^ HEK293T cells were incubated for 0.5, 1.0, and 3.0 h after resealing with 0.25 mg/ml of anti-IKKα antibody (clone Y463, Abcam, Cat# ab169743) or the same amount of normal rabbit antibody as a non-targeting negative control (NC). Resealing of anti-IKKα antibody led to a 40% reduction in IKKα protein levels within 30 min, which was further reduced by 65% in 3 h. Only a slight decrease in IKKβ was observed. Phosphorylation of NF-ĸB p65, downstream of IKKα, was also decreased under anti-IKKα-resealed conditions. Data expressed as mean ± SEM (*n* = 3). *p < 0.05, **p < 0.01, ns: not significant. Rb: rabbit, Ab: antibody. **(B)** Schematic of the IKKα Trim-Away experiment. **(C)** Western blotting results of HEK293T cells that underwent the same resealing procedure described in A were incubated for 3 h and were collected for analysis. Approximately 41% of the IKKα was degraded, which was limited compared with that in TRIM21-overexpressed Piranha^TM^ HEK293T cells. Data are expressed as mean ± SEM (*n* = 3). **P < 0.01, ns: not significant.

Then, we applied the cell resealing technique for Trim-Away-mediated protein degradation. Compared to capillary electroporation-mediated Trim-Away, which is the “gold standard” for protein degradation in bulk cell populations, cell resealing enables more rapid analysis because the cells can be kept adherent throughout the procedure. Given that TRIM21 is degraded together with the antibody–target complex, the ability to degrade the target is limited by the amount of endogenous TRIM21. Therefore, we used a HEK293T cell line constitutively expressing Piranha^TM^, a modified version of TRIM21 protein, to ensure that sufficient TRIM21 was supplied for target degradation. First, we tested the IkB kinase (IKK) IKKα, which is efficiently degraded by the conventional electroporation-mediated Trim-Away method (Clift et al., 2017). IKKα is one of the two catalytic subunits of the IKK complex and is a target involved in the activation of the nuclear factor kappa-Β (NF-ĸB) pathway (Figure 2B) (Hu et al., 1999; Kiyoshi et al., 1999; Clift et al., 2017). We resealed anti-IKKα antibody (or the same amount of normal rabbit antibody as a negative control) into the TRIM21-overexpressed Piranha^TM^ HEK293T cells. As shown in Figure 2A, resealing the anti-IKKα antibody led to a 40% reduction in IKKα protein levels within 30 min, which was further reduced to 65% at 3 h. The degradation efficiency was high until 12 h after resealing; however, after 24 h of incubation, IKKα levels recovered to some extent, which occurred simultaneously with the decrease in resealed antibody levels (Supplementary Figure S6). Interestingly, there was only a slight decrease in IKKβ, which forms the IKK complex together with IKKα, demonstrating that specific degradation was achieved using the anti-IKKα antibody/Trim-Away system (Figure 2A). Consistent with the results of siRNA-mediated IKKα suppression (Supplementary Figure S7), IKKα degradation by Trim-Away inactivated downstream signaling, as indicated by reduced phosphorylation of NF-ĸB p65 at serine 536 (Figure 2A) (Jiang et al., 2003). We confirmed that the degradation was proteasome-dependent by treating the cells with the proteasome inhibitor MG132, which inhibited IKKα degradation (Supplementary Figure S8). Further, we confirmed that antibody resealing itself did not alter target protein levels in all three cell lines (Supplementary Figure S9).

Next, we tested whether endogenous TRIM21 was sufficient for IKKα degradation by Trim-Away. We subjected HEK293T cells without TRIM21 overexpression to the same resealing procedure, and whole cell lysates were harvested 3 h later for western blotting. As expected, although the degree of IKKα degradation was limited compared with that in TRIM21-overexpressed Piranha^TM^ HEK293T cells, 41% of IKKα was degraded, and downstream phosphorylation of NF-ĸB p65 was also reduced (Figure 2C, Supplementary Figure S10). We confirmed that the observed degradation in HEK293T cells without TRIM21 overexpression was attributable to endogenous TRIM21 by siRNA-mediated knockdown (KD) of TRIM21 (Supplementary Figure S11). We also observed reduced phosphorylation of NF-ĸB p65 in TRIM21 KD cells, in which IKKα was not degraded, indicating that the anti-IKKα antibody (clone Y463) inhibits IKKα activity by binding to IKKα. This observation indicates that in Trim-Away experiments, intracellularly delivered antibodies exert an additional inhibitory effect by binding to as well as degrading the target protein, and this effect likely depends on the characteristics of the antibody (e.g., binding site and clonality). Such phenomena are common, or often intrinsic in targeted degradation strategies such as PROTACs. We also tested the effects of IKKα Trim-Away knockdown in HK-2 cells and HeLa cells (Supplementary Figures S12A,B); IKKα was successfully reduced in both the cell lines, showing that they had sufficient endogenous TRIM21 for Trim-Away degradation of IKKα.

### Improving the resealing protocol for a more versatile Trim-Away analysis system

**FIGURE 3:**
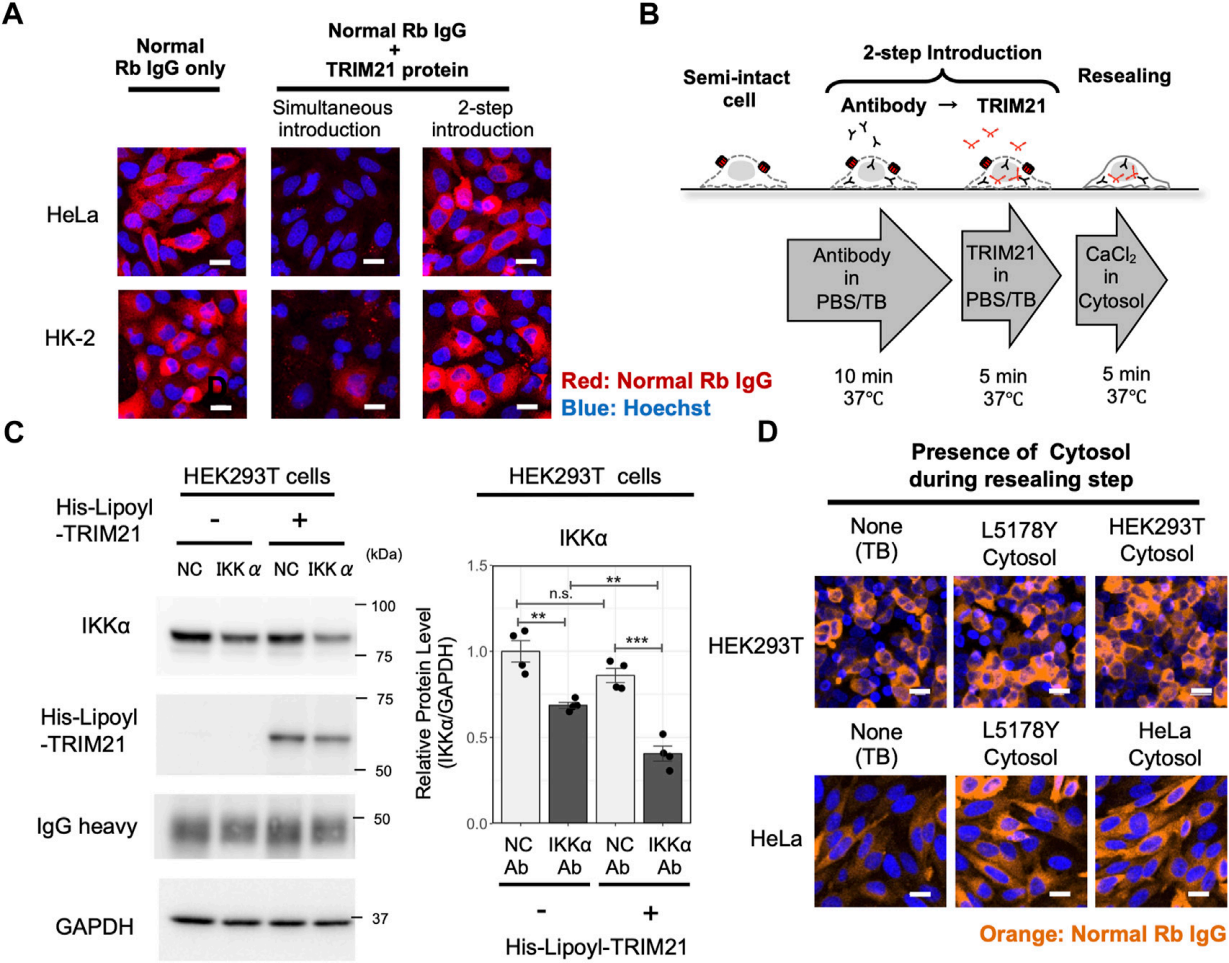
Establishment of a versatile Trim-Away analysis system by a two-step resealing protocol and a convenient cytosol preparation protocol. **(A)** Comparison of the antibody resealing efficiency between cells in which TRIM21 protein was introduced simultaneously with the antibody or by a two-step introduction protocol. HeLa cells or HK-2 cells were resealed with non-targeting normal rabbit IgG, after which they were incubated for 1 h and fixed for analysis. Efficient introduction of the antibody was observed in cells resealed by the two-step introduction protocol. Scale bar: 10 μm. Rb: rabbit. **(B)** Outline of the two-step introduction protocol. Cells were treated with ∼0.4 μg/ml of SLO on ice for 5 min and incubated with prewarmed transport buffer (TB) at 37°C for 5 min, thereby permeabilizing the cell membrane. Then, the semi-intact cells were incubated with antibody for 10 min, followed by incubation with TRIM21 protein for 5 min. Cells were then resealed by incubation with 1 mM CaCl_2_ for 5 min at 37°C and allowed to recover in the incubator at 37°C. **(C)** Western blotting showing enhanced degradation of IKKα by co-introduction of recombinant TRIM21 protein using the 2-step resealing protocol. HEK293T cells were incubated with 0.5 mg/ml of anti-IKKα antibody (clone Y463, Abcam, Cat# ab169743) or the same amount of normal rabbit antibody as a negative control (NC), followed by incubation with 6.5 mg/ml of TRIM21 protein, after which they were incubated for 3 h and collected for analysis. Resealing of anti-IKKα antibody alone showed a 31% reduction in IKKα protein levels, whereas resealing of anti-IKKα antibody and TRIM21 protein led to a 59% reduction. Data are expressed as mean ± SEM (*n* = 4). ***P < 0.001, ns: not significant. **(D)** Comparison of the antibody resealing efficiency between cells that were incubated without cytosol, with L5178Y cytosol, or with cytosol obtained by the new freeze-thaw protocol, during the resealing step. HEK293T cells or HeLa cells were resealed with normal rabbit IgG, after which they were incubated for 1 h and fixed for analysis. L5178Y cytosol and cytosol obtained by the new freeze-thaw protocol showed enhanced cell resealing efficiency compared to conditions resealed without cytosol. Scale bar: 20 μm.

Thus far, we confirmed the ability of cell resealing-mediated Trim-Away to degrade the target and inhibit downstream signaling. To expand the versatility of the method, we further aimed to improve some of the factors that might hinder its wide use. First, we sought to develop a resealing-mediated Trim-Away method that is applicable for cells with low TRIM21 levels. Although endogenous TRIM21 was sufficient for IKKα degradation in the three cell lines, the degree of IKKα degradation in HEK293T cells was lower than that in TRIM21-overexpressed Piranha^TM^ HEK293T cells (Figures 2A,C). As the burden of obtaining stable cells overexpressing TRIM21 or achieving transient expression of TRIM21 by transfection limits the broad application of Trim-Away, we next attempted to introduce the antibody together with recombinant TRIM21 protein by cell resealing. We purified functional recombinant TRIM21 using the protocol by Clift et al. (2017) (Supplementary Figure S13) and resealed the protein together with normal rabbit IgG. As shown in Figure 3A, Supplementary Figure S14, simultaneous introduction of the antibody and the TRIM21 protein resulted in a substantial decrease in the levels of antibodies delivered into the cytosol, presumably because the antibody–TRIM21 complex formed and aggregated outside the cells, making it difficult for it to pass through the SLO pores. Therefore, we established a two-step resealing protocol for efficient introduction of the antibody (see Figure 3A “Two-step introduction”) and the TRIM21 protein, as outlined in Figure 3B.

We then tested if we could enhance the degradation capacity of IKKα Trim-Away by the two-step resealing protocol. As shown in Figure 3C, Supplementary Figure S15, two-step resealing of the a-IKKα antibody and the TRIM21 protein in HEK293T cells enhanced the degradation rate; a 59% reduction in the IKKα level was observed with TRIM21 compared to a 31% reduction without TRIM21 supplementation. Thus, by supplementing TRIM21 protein in HEK293T cells, we could degrade IKKα to a similar extent as that in the TRIM21-overexpressed Piranha^TM^ HEK293T cell line.

Next, we examined the necessity of exogenous cytosol for antibody delivery and Trim-Away degradation, as the requirement of adding exogenous cytosol may be a major drawback of the cell resealing technique. As shown in Supplementary Figure S16A, the presence of exogenous cytosol enhanced the retention rate of the introduced antibody, indicating that the cytosol facilitated the efficient resealing of the membrane pores. In contrast, cells resealed in the absence of cytosol displayed higher susceptibility to cell death, as indicated by decreased cell counts and nuclear shrinkage, which may be due to inefficient resealing (Supplementary Figure S16A). Further examination revealed that efficient antibody resealing depended on the supplementation of the cytosol only during the resealing step (5 min incubation with calcium ions) and not during the antibody introduction step when SLO pores were present (Supplementary Figure S16B). Thus, although the conventional cell resealing protocol utilizes cytosol throughout the antibody introduction step and the resealing step, we concluded that cytosol was preferable only for the resealing step. As shown in Supplementary Figure S17, Trim-Away degradation of IKKα occurred without exogenous cytosol. However, we recommend supplementation with cytosol in the resealing step because efficient resealing helps the cells recover more rapidly and reduces cell death.

The conventional cell resealing protocol used cytosol taken from L5178Y cells, a murine lymphoma cell line that enables us to obtain a large amount of cytosol at once as they can be easily grown in suspension culture. However, it is preferable to use cytosol obtained from the same cell line. Thus, we established a new convenient protocol for obtaining cytosol by simple freeze-thaw and centrifugation. We tested the cell resealing efficiency using this cytosol, which showed similar efficiency for promoting cell resealing as the conventional L5178Y cytosol (Figure 3D and Supplementary Figure S18).

In the above experiments, we established an improved resealing protocol that efficiently delivers antibodies within 40 min with limited inflow of exogenous factors, followed by Trim-Away degradation, which enables rapid analysis within 3 h. The degradation capacity could be further increased by introducing recombinant TRIM21 protein with the two-step resealing protocol, reinforcing the versatility of the Trim-Away degradation system.

### Antibody screening using the cell resealing technique

A major disadvantage of Trim-Away is that it requires highly specific antibodies, which must be able to bind to the protein of interest under non-denaturing conditions. Although antibodies are commercially available for almost all proteins, only a fraction of such antibodies are appropriate for Trim-Away, emphasizing the need for validation of antibodies. When using delivery methods such as microinjection or electroporation, only one antibody can be intracellularly delivered at a time; thus, validation becomes laborious as the number of antibodies increases. In contrast, when using the cell resealing technique, antibody screening is more efficient because many antibodies can be assayed at once using standard multi-well plates and multi-channel pipettes.

**FIGURE 4:**
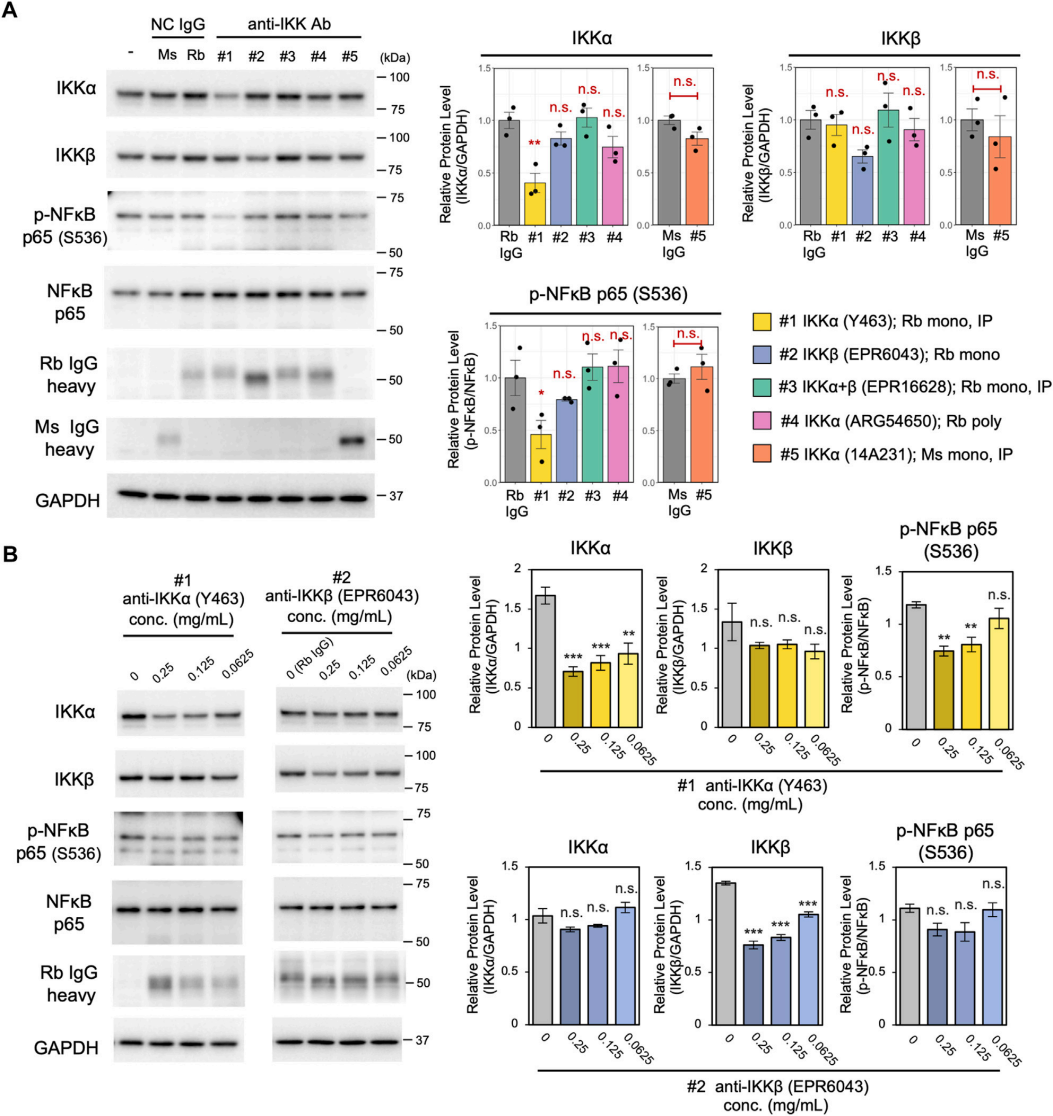
Screening of five different anti-IKK antibodies using the cell resealing technique. **(A)** TRIM21-overexpressed Piranha^TM^ HEK293T cells were resealed with 0.125 mg/ml of the respective anti-IKK antibodies and incubated for 3 h, and the efficiency and specificity of IKK degradation was tested by quantifying the degradation of IKKα and IKKβ using western blotting. Of the five tested antibodies, one anti-IKKα–specific antibody (clone Y463) significantly reduced IKKα levels. Although its effects were not significant, one anti-IKKβ–specific antibody (clone EPR6043) tended to reduce IKKβ levels. Data are expressed as the mean ± SEM (*n* = 3). For the four rabbit anti-IKK antibodies, the statistical significance of differences compared with the control (normal rabbit IgG) was assessed using Dunnett’s test as a multiple comparison test. For the single mouse anti-IKK antibody, Welch’s t-test was used to detect the difference compared with the control (normal mouse IgG). Rb: rabbit, Ms: mouse. **(B)** Concentration-dependent degradation ability of the two anti-IKK antibodies. The IKKα-specific antibody (clone Y463) degraded IKKα more efficiently than IKKβ, whereas the IKKβ-specific antibody (clone EPR6043) degraded IKKβ more efficiently than IKKα. The statistical significance of differences from the control (normal rabbit IgG) was assessed using Dunnett’s test. Data are expressed as mean ± SEM (*n* = 3). **P < 0.01, ***P < 0.001, ns: not significant.

Here, we applied the cell resealing technique to screen and assess the applicability for Trim-Away degradation of five different antibodies against IKK. TRIM21-overexpressed Piranha^TM^ HEK293T cells were resealed with 0.125 mg/ml of the respective anti-IKK antibodies and incubated for 3 h. Subsequently, the efficiency and specificity of IKK degradation was tested by quantifying the degradation of IKKα and IKKβ using western blotting. As shown in Figure 4A, only one of the five antibodies significantly degraded IKKα. Although not significant, one anti-IKKβ–specific antibody (clone EPR6043) tended to reduce IKKβ levels. Next, the two antibodies that displayed the highest potential for degrading IKKα and IKKβ, respectively, were further analyzed for their concentration-dependent degradation activity and specificity (Figure 4B). The IKKα-specific antibody (clone Y463) degraded IKKα more efficiently than IKKβ, whereas the IKKβ-specific antibody (clone EPR6043) degraded IKKβ more efficiently than IKKα. Both antibodies showed a concentration-dependent ability to degrade IKKα and IKKβ. Although we anticipated that antibodies recommended for immunoprecipitation by the manufacturer would be suitable for Trim-Away, the immunoprecipitation-validated antibodies for IKKα+β (clone EPR16628) and IKKα (clone 14A231) degraded neither IKKα nor IKKβ. Thus, the validation and selection of antibodies prior to conducting Trim-Away with the cell resealing technique is highly recommended to ensure the success of Trim-Away.

### Trim-Away-mediated degradation of mTOR *via* cytosolic antibody delivery using the cell resealing technique or adherent electroporation

**FIGURE 5:**
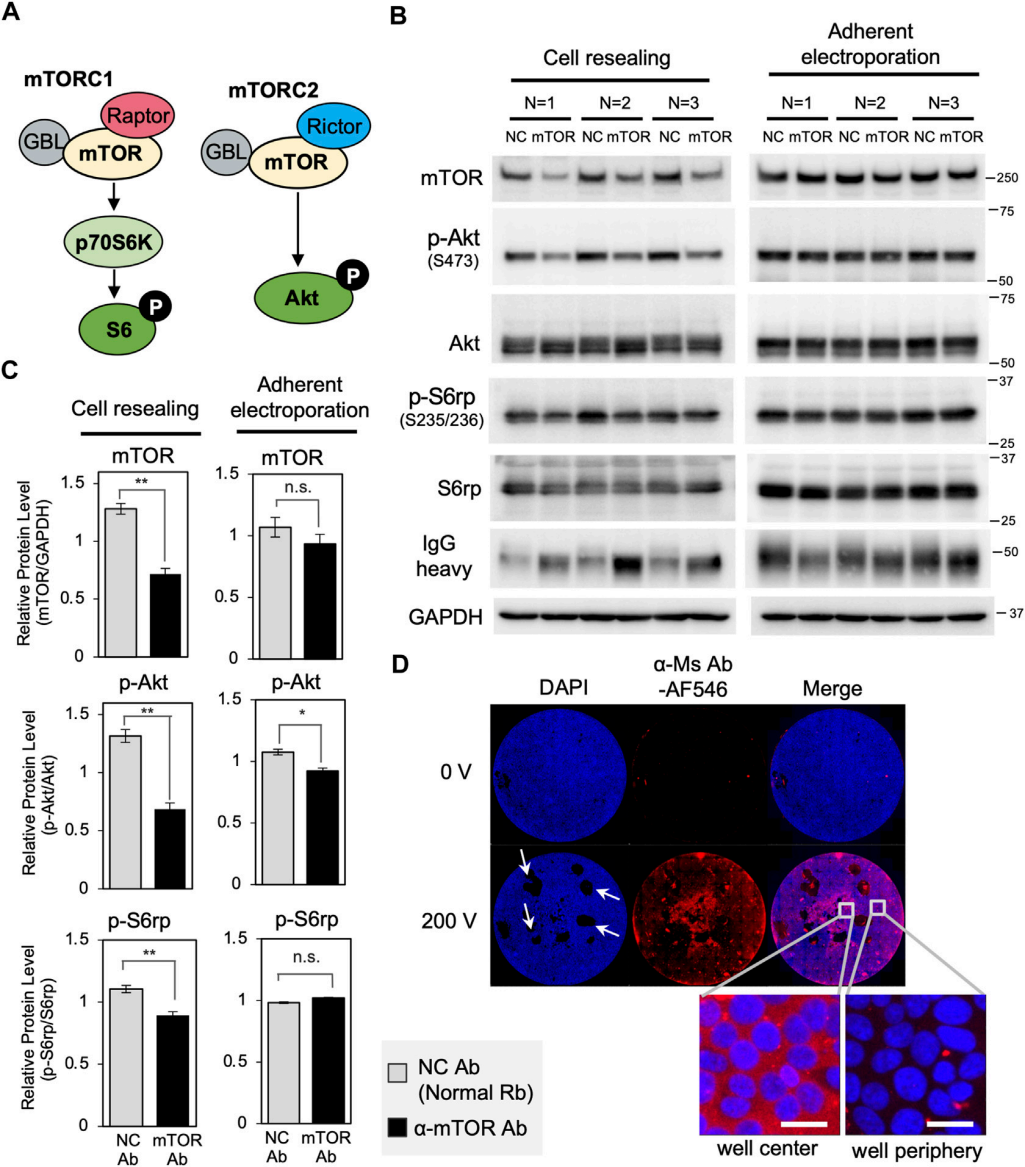
Trim-Away-mediated degradation of mTOR using the cell resealing technique or adherent electroporation. **(A)** Schematic of mTOR signaling. **(B)** The cell resealing technique or adherent electroporation were used to introduce anti-mTOR antibodies into TRIM21-overexpressed Piranha^TM^ HEK293T cells, which were then analyzed using western blotting. Cells were introduced with 0.5 mg/ml of anti-mTOR antibody (clone 7C10, CST, Cat#2983BF) or the same amount of normal rabbit antibody as a negative control (NC), after which they were incubated for 12 h and collected for analysis. **(C)** Bar graph showing quantification of the bands in (B). Resealing of anti-mTOR antibody led to a 44% reduction in mTOR protein level. Downstream signaling was also inactivated: phosphorylation of Akt at Ser473 and ribosomal protein 6 (S6rp) at Ser235/236 were reduced. Degradation of mTOR or inactivation of downstream signaling was not detected in the adherent electroporation-mediated experiment. Data are expressed as mean ± SEM (*n* = 3). *P < 0.05, **P < 0.01, ns: not significant. Rb: rabbit, Ab: antibody. **(D)** Microscopy images of Piranha^TM^ HEK293T cells with Alexa Fluor 546-labeled anti-mouse goat IgG (α-Ms Ab-AF546) introduced into the cells using adherent electroporation. Whole-well views show that only the cells near the center of the well that were located between the electrodes were well electroporated, whereas the majority of cells at the periphery were not electroporated. Scale bar: 20 μm. Ms: mouse.

Next, to test whether other target proteins were also degraded by the cell resealing-mediated Trim-Away system, we targeted mTOR, a central kinase regulating nutrient signaling and cell growth (Figure 5A) (Laplante and Sabatini, 2009; Clift et al., 2017) for degradation. We resealed 0.5 mg/ml of anti-mTOR antibody to TRIM21-overexpressed Piranha^TM^ HEK293T cells, resulting in a 44% reduction in mTOR protein level at 12 h after resealing (Figures 5B,C “Cell resealing”). Treatment with an mTOR-specific inhibitor, Torin, resulted in the inactivation of downstream signaling, i.e., reduced phosphorylation of Akt at S473 and ribosomal protein 6 (S6rp) at Ser235/236 (Supplementary Figure S19). Therefore, we assessed the inactivation of downstream signaling using mTOR Trim-Away, which revealed the suppression of this signaling (Figure 5, Supplementary Figure S20). Notably, endogenous TRIM21 was not sufficient to degrade the substantial amount of mTOR in HEK293T cells, whereas cells with additional expression of exogenous TRIM21 (“TRIM21-overexpressed HEK293T cells” in Supplementary Figure S21) showed improved mTOR degradation.

Comparing the degradation efficiency of mTOR with IKKα, the extent of mTOR degradation was less than that observed for IKKα, despite a higher amount of antibody used and a longer incubation time for degradation (comparing Figure 5C “Cell resealing” with Figure 2A). As the Trim-Away degradation efficiency depends on the amount of antibodies delivered to the cytosol, the amount of TRIM21 protein, and the endogenous level of the target protein, the amount of antibody and TRIM21 protein may have been insufficient to degrade the high abundance of mTOR. Another possible reason for the lower degradation efficiency might derive from the inaccessibility of the antibody to the target. A subset of mTOR, which is known to localize to lysosomes (Betz and Hall, 2013), might have been difficult to target for degradation (Clift et al., 2017).

Here, we also tested adherent electroporation-mediated antibody delivery using electrodes designed for 96-well plates. As with cell resealing, adherent electroporation allows rapid analysis following antibody introduction by maintaining cell adherence throughout the procedure, in contrast to capillary electroporation or conventional cuvette electroporation. Therefore, we aimed to compare the efficiency of adherent electroporation-mediated Trim-Away with that of cell resealing-mediated Trim-Away. After determining the optimal pulse conditions for adherent electroporation-mediated antibody delivery (Supplementary Figure S22), we introduced anti-mTOR antibody through adherent electroporation and analyzed Trim-Away-mediated degradation using western blotting. We used 0.5 mg/ml of anti-mTOR antibody for adherent electroporation-mediated antibody delivery; this concentration was the same as that used in the resealing experiment. As shown in Figures 5B,C (“Adherent electroporation”), we did not detect significant degradation of mTOR or the inhibition of downstream signaling. This is likely because only the cells near the center of the well, which were located between the electrodes, were well electroporated, whereas the vast majority of cells at the periphery were not well electroporated (Figure 5D). In contrast to adherent electroporation, the cell resealing technique enables the delivery of antibodies into most of the cells in the well, whether they are at the center or the periphery (see the whole-well view in Figure 1C). Thus, in comparing the two antibody introduction methods for adherent cells, only the cell resealing technique was suitable for Trim-Away when analyzed by western blotting.

### Multiplex immunofluorescence enables single-cell analysis for Trim-Away

Considering the advantage of the cell resealing technique, which enables antibody introduction while keeping the cells adherent, microscopic image-based single-cell analysis is especially suitable for detecting and analyzing Trim-Away. In addition to the ability to detect the morphology of cells or the localization of proteins, another important advantage of microscopic analysis is the ability to compensate for the cell-to-cell variation in the amount of resealed antibody. As there are cell-to-cell differences in membrane cholesterol levels that affect the number of SLO molecules that can bind to the membrane cholesterol to form pores, cell-to-cell variation in the amount of resealed antibody is inevitable. Accordingly, in analyses such as western blotting, which use whole cell lysates, the results represent the average of a heterogenous cell population, i.e., in some cells antibodies are abundant, whereas in other cells they are scarce. In contrast, microscopic single-cell analysis enables a more sensitive and precise analysis by examining only the efficiently resealed cells or the correlation between the amount of antibody introduced and the degradation efficiency. We have employed single-cell analysis with immunofluorescence and confocal microscopy in our previous studies, and this technique enabled the successful analysis and phenotyping of various cells, including resealed cells (Kano et al., 2017; Kunishige et al., 2020; Noguchi et al., 2021). Therefore, we reasoned that single-cell analysis would allow us to detect the effects of Trim-Away more clearly.

Immunofluorescence is a standard method for detecting the morphology of cells as well as the amount, modification state, and localization of proteins in a single-cell. However, conventional immunofluorescence imaging is limited to 4–6 channels, making it difficult to analyze the complex relationships between proteins in heterogeneous cell populations. As such, we adopted cyclic immunofluorescence, a method used to produce highly multiplexed imaging *via* a repeated staining and bleaching procedure, which can be conducted using standard reagents and microscopes (Lin et al., 2015, 2016; Radtke et al., 2020, 2022). Here, we established a cyclic immunofluorescence single-cell analysis system by optimizing the staining and bleaching protocol as well as the image acquisition procedure for obtaining the same field of view and the alignment of the images obtained from different cycles. By combining Trim-Away with cyclic immunofluorescence, we were able to simultaneously detect protein degradation and the resulting perturbation of downstream signal transduction at the single-cell level.

**FIGURE 6:**
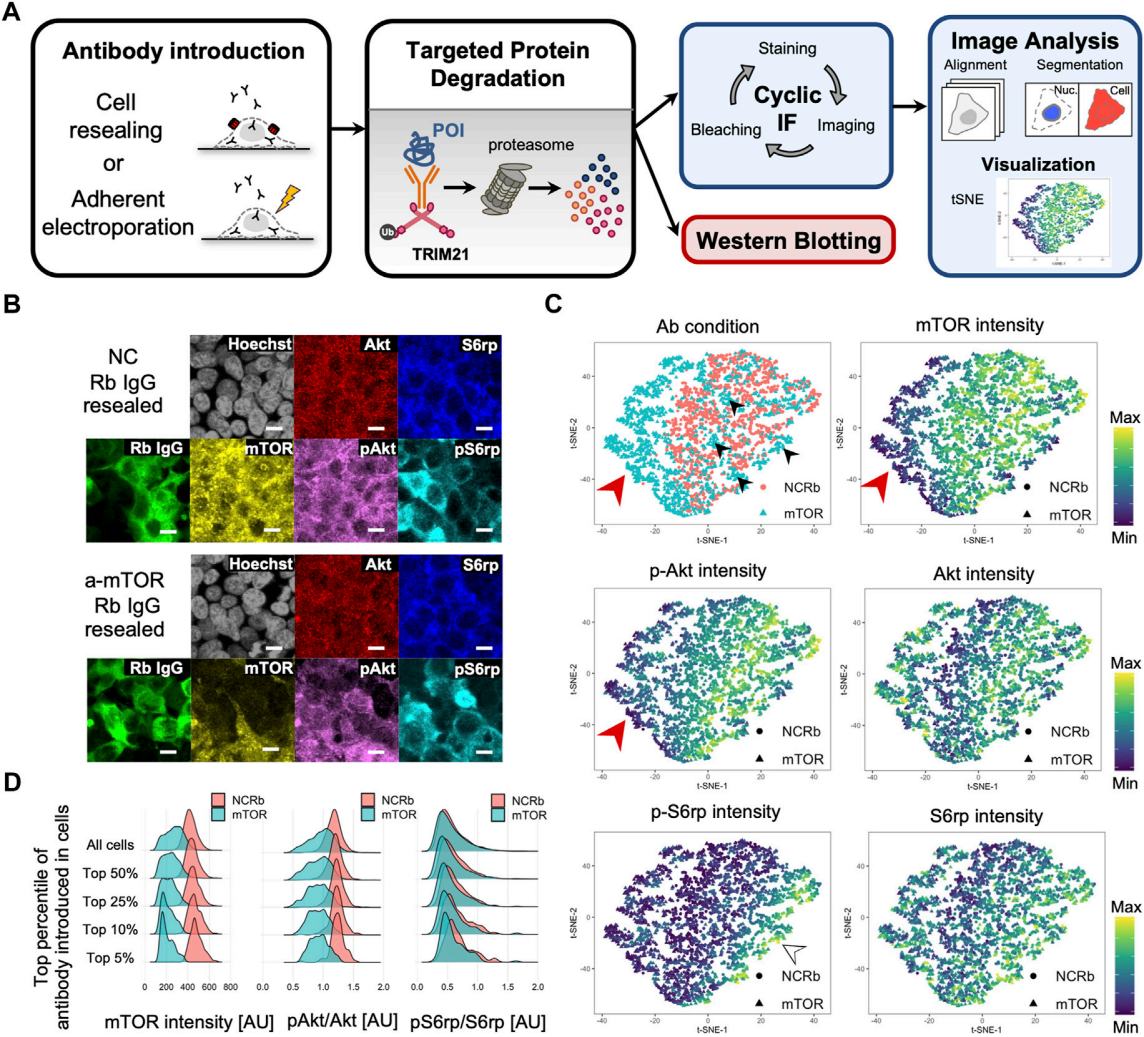
Single-cell analysis of cell resealing-mediated Trim-Away targeting mTOR. **(A)** Outline of the Trim-Away experiment followed by cyclic immunofluorescence and single-cell analysis. First, Trim-Away was performed with TRIM21-overexpressed Piranha^TM^ HEK293T cells grown on 96-well plates by introducing anti-protein of interest antibodies. After incubation, the cells were fixed, permeabilized, and stained with the first set of antibodies, after which they were automatically imaged using an A1 confocal microscope and NIS-Elements software. After imaging, the cells were bleached using hydrogen peroxide, high pH, and light exposure, and a second round of staining was conducted *via* direct immunofluorescence using Alexa Fluor-labeled antibodies. After repeated rounds of immunofluorescence staining, imaging, and bleaching, all images were aligned and analyzed. For the mTOR Trim-Away experiment, three cycles of cyclic immunofluorescence were performed: DAPI, rabbit antibody (mTOR antibody or normal rabbit antibody), and S6rp were stained for the first cycle; DAPI, phosphorylated S6rp, and mTOR were stained for the second cycle; and DAPI, Akt, and phosphorylated Akt were stained for the third cycle. Mean intensities of mTOR, phosphorylated Akt, Akt, phosphorylated S6rp, S6rp, rabbit antibody (mTOR antibody or normal rabbit antibody), and Hoechst were quantified per cell using confocal images. **(B)** Confocal microscope images of TRIM21-overexpressed Piranha^TM^ HEK293T cells that were resealed with anti-mTOR antibody (clone 7C10, CST, Cat#2983BF) or the same amount of normal rabbit antibody as a negative control (NC). Cells were incubated for 12 h and underwent the cyclic immunofluorescence procedure outlined in (A). Cells with anti-mTOR antibodies tended to have lower levels of mTOR and phosphorylated Akt. Scale bar: 10 μm. NC: negative control, Rb: rabbit. **(C)** Single-cell multidimensional data were visualized using the dimensionality reduction algorithm t-distributed stochastic neighbor embedding (t-SNE). The top left panel is color-coded by the antibody condition of each cell, i.e., anti-mTOR antibody or normal rabbit IgG (NCRb); a distinguishable domain consisting of anti-mTOR antibody-resealed cells is shown on the left (see the red arrowhead). All other maps are color-coded by the mean intensity of the respective proteins. The mTOR map (top right panel) and pAkt map (middle left panel) showed similar fluorescence intensity patterns, indicating that cells with high levels of mTOR had high levels of phosphorylated Akt. **(D)** Density plots showing mTOR intensity, pAkt/Akt, and pS6rp/S6rp of cells resealed with mTOR or normal rabbit IgG (NCRb). Single-cell data were filtered by the top percentile of the fluorescence intensity of the introduced antibody. The difference in distribution between anti-mTOR-resealed cells and the negative control became more pronounced as the data were narrowed down to the most efficiently resealed cells. AU: arbitrary unit.

As outlined in Figure 6A, we first performed mTOR Trim-Away *via* cell resealing, and this was followed by fixation and three cycles of cyclic immunofluorescence (as described in the Experimental Procedures). The mean intensities of mTOR, phosphorylated Akt (pAkt), Akt, phosphorylated S6rp (pS6rp), S6rp, rabbit antibody (mTOR antibody or normal rabbit antibody), and Hoechst were quantified per cell using confocal images (Figure 6B).

For single-cell data analysis, we first created pairwise plots from the multivariate data, which enabled us to explore distributions and correlations (Supplementary Figure S23). A histogram of fluorescence intensity indicated that the anti-mTOR antibody resealing condition had lower mTOR and pAkt intensities. In addition, a scatterplot showing the fluorescence of resealed anti-mTOR antibody and the immunofluorescence-stained mTOR fluorescence revealed a weak negative correlation between the two, indicating that cells with higher amounts of mTOR antibodies were able to degrade more mTOR. The same tendency was detected for pAkt and the resealed anti-mTOR antibody. For comparison, we applied the cyclic immunofluorescence analysis framework described above for the single-cell analysis of adherent electroporation-mediated Trim-Away. In contrast to the western blotting results, we were able to detect Trim-Away degradation of mTOR and its downstream effects by microscopic image-based single-cell analysis, which enabled us to focus only on the well electroporated cells (Supplementary Figure S24).

In further analyses, we applied t-distributed stochastic neighbor embedding (t-SNE) (Van der Maaten and Hinton, 2008), a dimensionality reduction algorithm useful for visualizing high-dimensional single-cell data in a two-dimensional space (Figure 6C) (Amir et al., 2013; Lin et al., 2015, 2016). We analyzed cells in which negative control antibodies or anti-mTOR antibodies had been delivered, and we distinguished a domain on the left side of the plot consisting of anti-mTOR antibody-resealed cells (see the red arrowhead in Figure 6C “Ab condition”). However, some of the cells in the anti-mTOR resealed condition diffused outside this domain; presumably, these cells were insufficiently permeabilized (see the black arrowheads in Figure 6C “Ab condition”). Importantly, the anti-mTOR antibody-resealed cells clustered on the left side of the plot had relatively low mTOR and pAkt intensity levels (see the red arrowhead in Figure 6C “mTOR” and “pAkt” plots). Consequently, we reasoned that the cells with higher amounts of mTOR antibodies were able to degrade more mTOR and that the downstream phosphorylation of Akt and S6rp would likely be reduced. Accordingly, we filtered cells that were in the top 5%, 10%, 25%, and 50% by the fluorescence of the introduced antibody. Figure 6D includes density plots showing the mean intensity of mTOR and phosphorylated levels of Akt and S6rp. As expected, the difference in the density plots between anti-mTOR and the negative control became clearer as the data were narrowed down to the most efficiently resealed cells. The same tendency was observed for phosphorylated levels of Akt and S6rp (see Figure 6D “pAkt/Akt” and “pS6/S6rp”).

Thus, the cyclic immunofluorescence method and subsequent microscopic image-based single-cell analysis were found to be a suitable analysis system for cell resealing-mediated Trim-Away, where cell-to-cell variation in the amount of antibody had been introduced.

## Discussion

Trim-Away-mediated targeted protein degradation is a promising technique for studying endogenous protein function. Previous studies have mainly used capillary electroporation and microinjection for cytosolic antibody delivery; however, these methods have low throughput and require the use of special skills and/or equipment, so they have hindered the widespread use of Trim-Away. In this study, we used a cell resealing technique for cytosolic antibody delivery; this simple, high-throughput method does not require the use of special skills or equipment. Compared with capillary electroporation, which results in a near complete depletion of the target protein (Clift et al., 2017), cell resealing exhibited modest degradation efficiency when assessed using western blotting because of cell-to-cell variation in resealing efficiency. However, the resealing-mediated method has its own advantage; that is, because the cells are kept adherent throughout the resealing procedure, the polarity of the cells is maintained, which is an important aspect for epithelial cells and neuronal cells. In addition, this new approach enabled us to take full advantage of the rapid Trim-Away degradation system by rapidly analyzing the cells into which antibodies were delivered (see Supplementary Table S1 for comparison of antibody introduction methods). We demonstrated antibody resealing and subsequent Trim-Away degradation of IKKα in three cell lines (HEK293T, HeLa, and HK-2 cells). Furthermore, western blotting analysis revealed the rapid degradation of the target IKKα, which was significantly degraded within 30 min following anti-IKKα antibody resealing.

While the cell resealing technique has several advantages over other antibody delivery methods, some limitations in the conventional resealing technique, such as the requirement of exogenous cytosol and the cell-to-cell variation in resealing efficiency, have presumably hindered its application to Trim-Away. Therefore, we have established a versatile cell resealing-mediated Trim-Away analysis system by optimizing the conventional resealing protocol and applying microscopic image-based single-cell analysis. First, we developed a simplified protocol in which exogenous cytosol is supplemented only in the cell resealing step. We revealed that exogenous cytosol was not necessary for the antibody introduction step but was preferred for the subsequent cell resealing step to enhance the retention of the introduced antibody. Using this simplified protocol, the inflow of exogenous cytosol can be minimized, which reduces the risk of perturbing the physiology of the host cell. Furthermore, we developed a more convenient, small-scale cytosol preparation method. This method enables us to easily obtain cytosol from the same cell line as the host cell, which simplifies the experimental conditions and facilitates the interpretation of the results from the resealing experiments. Although these improvements can largely reduce the obstacles to using the cell resealing technique, it is more preferable to use a defined solution rather than cytosol. Thus, investigation of the specific cytosolic element(s) that facilitate membrane resealing would contribute to further improvement of the resealing protocol. Annexins may be the leading candidates, as they are known to promote membrane repair in membrane-injured cells, including SLO-perforated cells (Babiychuk et al., 2009, 2011; Demonbreun et al., 2019). Second, we developed a two-step resealing protocol for the co-introduction of the antibody and recombinant TRIM21 protein, thereby enhancing the degradation capacity. This two-step resealing protocol enables the application of Trim-Away in cells with limited TRIM21 protein levels. Third, we applied cyclic immunofluorescence and microscopic image-based single-cell analysis to handle the cell-to-cell variation of the resealed cells. Although the cell resealing technique enabled the delivery of antibodies into most cells throughout the well, some heterogeneity existed in terms of the quantity of resealed antibodies; this was derived from the cell-to-cell variance in permeabilizing and/or resealing efficiency. Thus, we attempted to compensate for this cell-to-cell variance using quantitative single-cell analysis conducted *via* cyclic immunofluorescence and microscopic image-based analysis. Consequently, we were able to reduce the number of samples required to analyze all variables (i.e., stained proteins). Moreover, cyclic immunofluorescence enabled simultaneously detection of multiple proteins in single cells. We visualized the multidimensional data using the dimensionality reduction algorithm t-SNE (Van der Maaten and Hinton, 2008); hence, we were able to easily comprehend the complex data derived from heterogeneous cell populations. Indeed, by visualizing the single-cell data obtained from our Trim-Away experiment targeting mTOR (Figure 6C), we observed that the fluorescence intensity patterns for mTOR and pAkt were similar, indicating that cells with high levels of mTOR had high levels of phosphorylated Akt. In contrast to mTOR or pAkt, there appeared to be a distinct cell population with high levels of pS6rp (see the white arrowhead in Figure 6C “pS6rp” plots), indicating that a factor other than mTOR abundance affected the phosphorylation level of S6rp, e.g., the cell cycle (Gabriele et al., 2018). Although we detected only six proteins using cyclic immunofluorescence, the duration of this multiplex approach can be easily extended by increasing the number of cycles to detect more proteins. Visualization using t-SNE or another dimensionality reduction algorithm becomes vital as dimensionality increases; such algorithms help researchers extract information from the complex data that comes from heterogeneous cell populations. Overall, we strongly recommend using microscopic image-based quantitative analysis, especially multiplex analysis with cyclic immunofluorescence, to evaluate the results of cell resealing-mediated Trim-Away.

The major challenge inherent in Trim-Away is its need for highly specific antibodies that can to bind to the protein of interest under non-denaturing conditions. Antibodies are available for most proteins, but not all can be used with Trim-Away. Importantly, we were able to use the cell resealing technique to screen and assess five different antibodies against IKK for Trim-Away, finding that two of the five antibodies efficiently degraded the target. Surprisingly, even antibodies that were recommended for immunoprecipitation by the manufacturer and were expected to bind to the target protein in its native conformation in cells were not necessarily suitable for Trim-Away, emphasizing the need for screening and assessment of Trim-Away-specific antibodies. Unlike other low-throughput delivery methods, such as microinjection or electroporation, the cell resealing technique enables the validation of many antibodies at once. Thus, the reported technique enables simple and practical antibody validation that can be used for antibody selection prior to a Trim-Away experiment.

Of note, whereas protein targets that shuttle in and out of the nucleus can be targeted for Trim-Away, proteins that are retained in the nucleus cannot be targeted by conventional antibodies unless they are directly delivered into the nucleus (e.g., microinjection). However, this limitation can be overcome by utilizing an Fc-domain-fused nanobody, which is small enough to enter the nucleus. Indeed, Clift et al. (2017) demonstrated the degradation of H2B-GFP by co-expressing TRIM21 and the Fc-nanobody fusion. This approach using Fc-nanobody fusion to target proteins inside the nucleus should work for the cell resealing technique as well.

Trim-Away requires large quantities of antibodies, which is a possible hurdle for the adoption of the method. For example, 10 μg of antibody was required per well for IKKα Trim-Away, even in a 96-well plate format. In our resealing experiment, we noted that it was possible to reduce the consumption of antibodies by collecting the antibody mixture and reusing it in another resealing experiment. Using Trim-Away to target mTOR, we found that the degradation ability of the anti-mTOR antibody mixture was not attenuated throughout three cycles of resealing-mediated Trim-Away (Figure 5B “Cell resealing”). Such reusing of antibodies would be difficult when using other delivery methods, including capillary electroporation, which requires cell suspension; in such methods, excess antibodies are used for a single antibody delivery procedure in which only a fraction of the antibodies are introduced, while the rest are washed away. Thus, along with the rapid analysis capabilities of antibody delivery methods for adherent cells including cell resealing, the reusability of antibodies is another advantage.

For comparison with the cell resealing-mediated Trim-Away method, we also applied adherent electroporation, which is a simple process requiring only a few minutes for antibody introduction. In contrast to cell resealing, Trim-Away degradation mediated by adherent electroporation was not detectable with western blotting analysis because only the cells located between the electrodes were efficiently electroporated. Similarly, other analysis methods that require the use of whole cell lysates, e.g., quantitative PCR and ELISA, would be unsuitable for adherent electroporation. However, we were able to detect Trim-Away degradation by microscopic image-based single-cell analysis, demonstrating the versatility of this analysis approach.

Recently, targeted protein degradation has gained increasing attention as a novel therapeutic modality that could target the undruggable proteome (Singh et al., 2019; Luh et al., 2020; Yang et al., 2020). In particular, hydrophobic tagging (Neklesa et al., 2011; Neklesa and Crews, 2012), molecular glue (Chamberlain and Hamann, 2019), and PROTACs (Sakamoto et al., 2001) have great potential for clinical application, and two PROTAC degraders are currently being tested in phase II trials (Békés et al., 2022). In contrast to these methods using small molecule degraders, cell resealing-mediated Trim-Away is unavailable for *in vivo* applications. However, as chemical biology tools for exploring protein functions, small molecules approaches may not be easily accessible to several biologists because they are dependent on the identification of a suitable target binder; moreover, the degrader molecules must be designed for each protein of interest. A more versatile approach that does not require a dedicated target binder is to fuse a protein tag to the target protein and then degrade the protein through selective recognition of the attached tag. Approaches based on protein tags include Halo Tags (Buckley et al., 2015; Tomoshige et al., 2015), dTag system (Nabet et al., 2018), degrons such as auxin-inducible degrons (Yesbolatova et al., 2020), and small molecule-assisted shutoff (Chung et al., 2015). Although applicable to a wider range of targets, these degradation systems require modification of the genome for attaching the tag, and it is necessary to verify that no undesired alterations are caused by attaching the tags. Compared with the aforementioned approaches, Trim-Away does not require prior modification of the genome, and there is no need to design a degrader molecule for each protein of interest. Additionally, although the reliance on E3 ligases for targeted protein degradation has been one of the main issues for cells with low levels of E3 ligases, Trim-Away can be used in such cells by supplementing it with TRIM21 protein. Furthermore, in contrast to E3 ligases used in PROTACs, such as cereblon or von Hippel–Lindau, TRIM21 does not have a cellular housekeeping function; therefore, it is less likely to interfere with normal metabolism by competing with endogenous substrates (Cromm and Crews, 2017; Clift et al., 2018). A major barrier to Trim-Away has been the limited means for delivering intracellular antibodies, which we aimed to overcome in this study. Another barrier may be that Trim-Away is dependent on selective antibodies against the protein of interest, and there may be no suitable antibodies available for some proteins, especially minor proteins. Overall, when a degrader molecule is already available for the target protein, target protein degradation can be achieved most simply by treating the cells with the molecule. Otherwise, approaches using protein tags or Trim-Away could be a promising alternative, although both methods require more extensive manipulation of the cells. When targeting minor proteins without suitable antibodies, approaches using protein tags would be a better choice. Conversely, when targeting proteins for which attaching a tag interferes with their function, Trim-Away would be a more suitable approach. Consequently, we expect that the complementary use of these techniques will enable the targeting of a wide variety of proteins for degradation.

Until recently, Trim-Away has only been proposed for use in cellular systems. However, some carrier-mediated antibody delivery methods have been developed and applied for Trim-Away that have the potential for *in vivo* applications (Du et al., 2020; Safitri et al., 2021; Sui et al., 2021). In addition, the use of cell-penetrating polymers modified with tumor-targeting ligands enables the specific targeting of cancer cells for Trim-Away protein knockdown. Thus, carrier-mediated antibody delivery methods are most likely to be used in future *in vivo* applications and for clinical translation. However, for basic research purposes, such as studying endogenous protein functions and cellular pathways, the gradual introduction of antibodies by endocytosis (or other pathways) might be a drawback. Cell-to-cell differences in uptake activity make it difficult to interpret phenotypes because those detected are a mixture of the direct and secondary consequences of Trim-Away protein knockdown. Consequently, methods that enable the instant delivery of antibodies, such as cell resealing or electroporation, are more relevant for protein function studies. In addition, although cell resealing is not amenable to *in vivo* applications, it has a great potential for validating therapeutic targets or high-throughput screening of therapeutic antibody drugs.

In terms of future applications, cell resealing-mediated Trim-Away could be used for protein function studies in disease model cells by coupling with cell resealing-mediated recreation of disease model cells. The cell resealing technique can be used to not only introduce several types of molecules at once but also to exchange the whole cytosol and manipulate/synchronize the intracellular environment (Kano et al., 2005, 2017). Indeed, we have previously demonstrated that a diabetic or healthy model cell can be recreated using normal HeLa cells or hepatic H4IIEC3 cells by introducing disease-related or healthy cytosol obtained from the liver of diabetic (e.g., db/db) or healthy mice, respectively (Kano et al., 2017). The study of disease model cells helps elucidate pathological perturbations in cellular function. Targeted protein degradation *via* the Trim-Away method would be another useful strategy for studying pathological cellular states; it could be coupled easily with the recreation of disease model cells by simultaneously resealing pathological cytosol with the antibody against the protein of interest.

In summary, we developed a versatile cell resealing technique for the intracellular delivery of antibodies and targeted protein degradation through Trim-Away. Antibodies could be introduced readily into adherent cells, enabling rapid analysis through both western blotting and immunofluorescence analysis. Furthermore, the use of microscopic image-based analysis, cyclic immunofluorescence, and t-SNE allowed us to comprehend the complex data derived from heterogeneous cell populations. When combined with single-cell multiplex analysis, cell resealing-mediated Trim-Away represents a promising method for studying endogenous protein function.

## Data Availability

The original contributions presented in the study are included in the article/Supplementary Material; further inquiries can be directed to the corresponding author.
