# Supplementary material for: Targeted protein degradation by Trim-Away using cell resealing coupled with microscopic image-based quantitative analysis
Source: Front Cell Dev Biol. 2022 Dec 19;10:1027043. doi: 10.3389/fcell.2022.1027043 (PMC9806799; doi:10.3389/fcell.2022.1027043)
Supplement: Supplementary file 1 [file DataSheet1.pdf]

Supplementary Material

1. Supplementary Figures

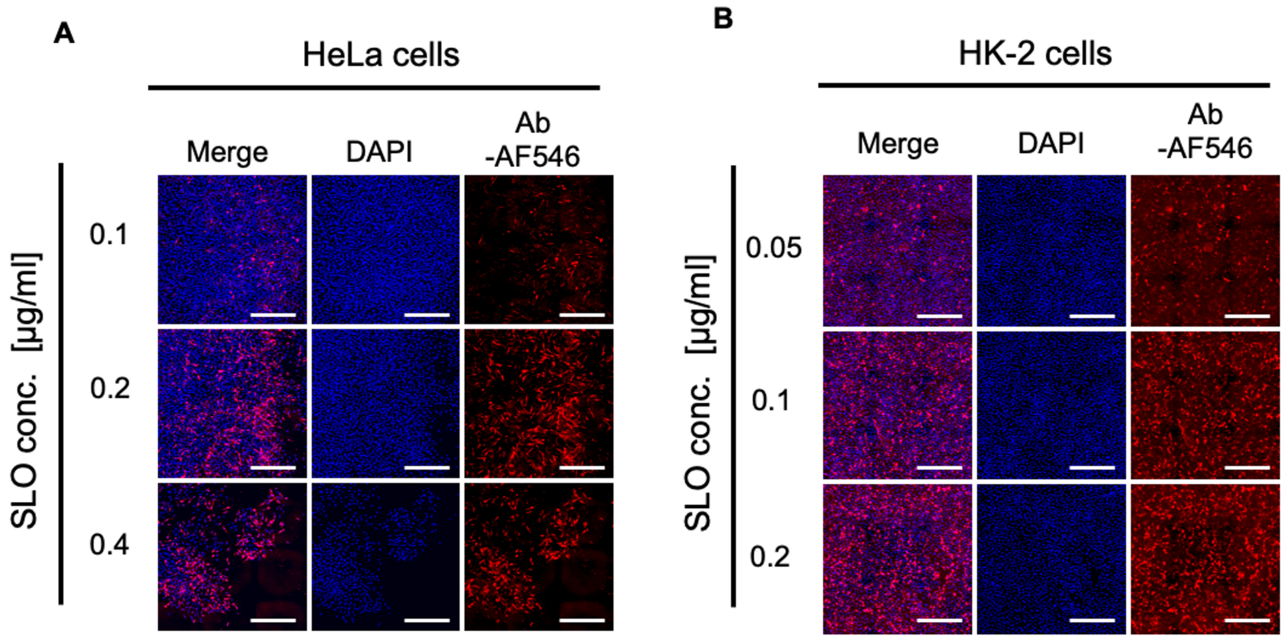

Fig. S1 Evaluation of the optimal streptolysin O (SLO) concentration in HeLa and HK-2 cells.

(A) Evaluation of optimal SLO concentration in HeLa cells. Cells were permeabilized with 0.1–0.4 μg/mL of SLO, and Alexa Fluor 546-labeled antibodies were resealed according to the protocol outlined in Fig. 1A. Cells were fixed 3 h later and imaged using Cytation 5. The optimal SLO concentrations were 0.2 μg/mL. Scale bar: 500 μm.

(B) Evaluation of optimal SLO concentration in HK-2 cells. Cells were permeabilized with 0.05–0.2 μg/mL of SLO, and Alexa Fluor 546-labeled antibodies were resealed according to the protocol outlined in Fig. 1A. Cells were fixed 3 h later and imaged using Cytation 5. The optimal SLO concentrations were 0.2 μg/mL. Scale bar: 500 μm.

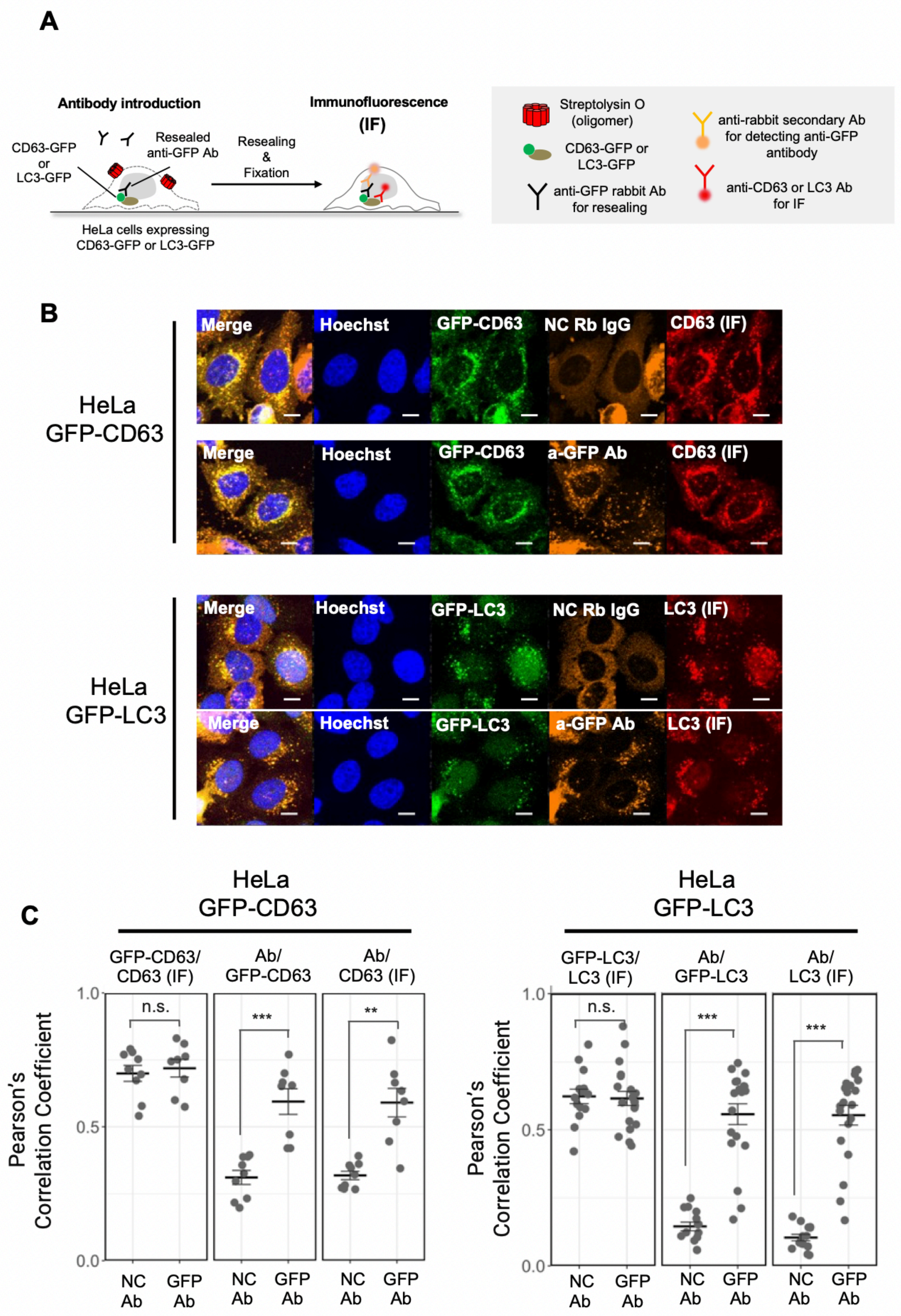
A
Antibody introduction
Immunofluorescence (IF)
CD63-GFP or LC3-GFP
Resealed anti-GFP Ab
Resealing & Fixation
HeLa cells expressing CD63-GFP or LC3-GFP
Streptolysin O (oligomer)
CD63-GFP or LC3-GFP
anti-GFP rabbit Ab for resealing
anti-rabbit secondary Ab for detecting anti-GFP antibody
anti-CD63 or LC3 Ab for IF
B
HeLa GFP-CD63
Merge
Hoechst
GFP-CD63
NC Rb IgG
CD63 (IF)
a-GFP Ab
HeLa GFP-LC3
GFP-LC3
LC3 (IF)
C
HeLa GFP-CD63
HeLa GFP-LC3
GFP-CD63/ CD63 (IF)
Ab/ GFP-CD63
Ab/ CD63 (IF)
GFP-LC3/ LC3 (IF)
Ab/ GFP-LC3
Ab/ LC3 (IF)
Pearson's Correlation Coefficient
n.s.
***
**
1.0
0.5
0.0
NC Ab
GFP Ab

Fig. S2 Evaluation of the targeting ability of resealed antibodies.

(A) Process for evaluating the targeting ability of resealed antibodies. HeLa cells expressing CD63-GFP or LC3-GFP were resealed with anti-GFP rabbit antibody and subjected to immunofluorescence. Anti-rabbit IgG secondary antibody labeled with Alexa Fluor 546 was used to detect the resealed anti-GFP antibody. Anti-CD63 or LC3 mouse antibody was used for immunofluorescence; it was detected with anti-mouse IgG secondary antibody labeled with Alexa Fluor 647.

(B) Confocal microscopy images showing the targeting ability of resealed anti-GFP antibodies. Resealed anti-GFP antibodies (orange) showed punctate patterns and colocalization with GFP-tagged proteins (green). Immunofluorescence using antibodies against the exogenously expressed proteins (red) also showed colocalization of the two protein types. Normal rabbit IgG was used as a nontargeting negative control ("NC Rb IgG"), showing a diffused pattern. Scale bar: 10 μm.

(C) Quantifications of the colocalization level of images (B). Pearson's correlation coefficient between the indicated channels was calculated using the pixel intensity of the cytosol region for each cell. Each dot represents the value of one cell, and the bar indicates the mean ± standard error of the mean. Anti-GFP antibody (a-GFP Ab) displayed higher colocalization with GFP fused proteins (GFP-CD63 or GFP-LC3) as well as with the target proteins detected via immunofluorescence (CD63(IF) or LC3(IF)) compared with negative control antibodies (NC Rb IgG). Statistical significance of differences between the two groups was detected using Welch's t-test. $^{**}P < 0.01$, $^{***}P < 0.001$.

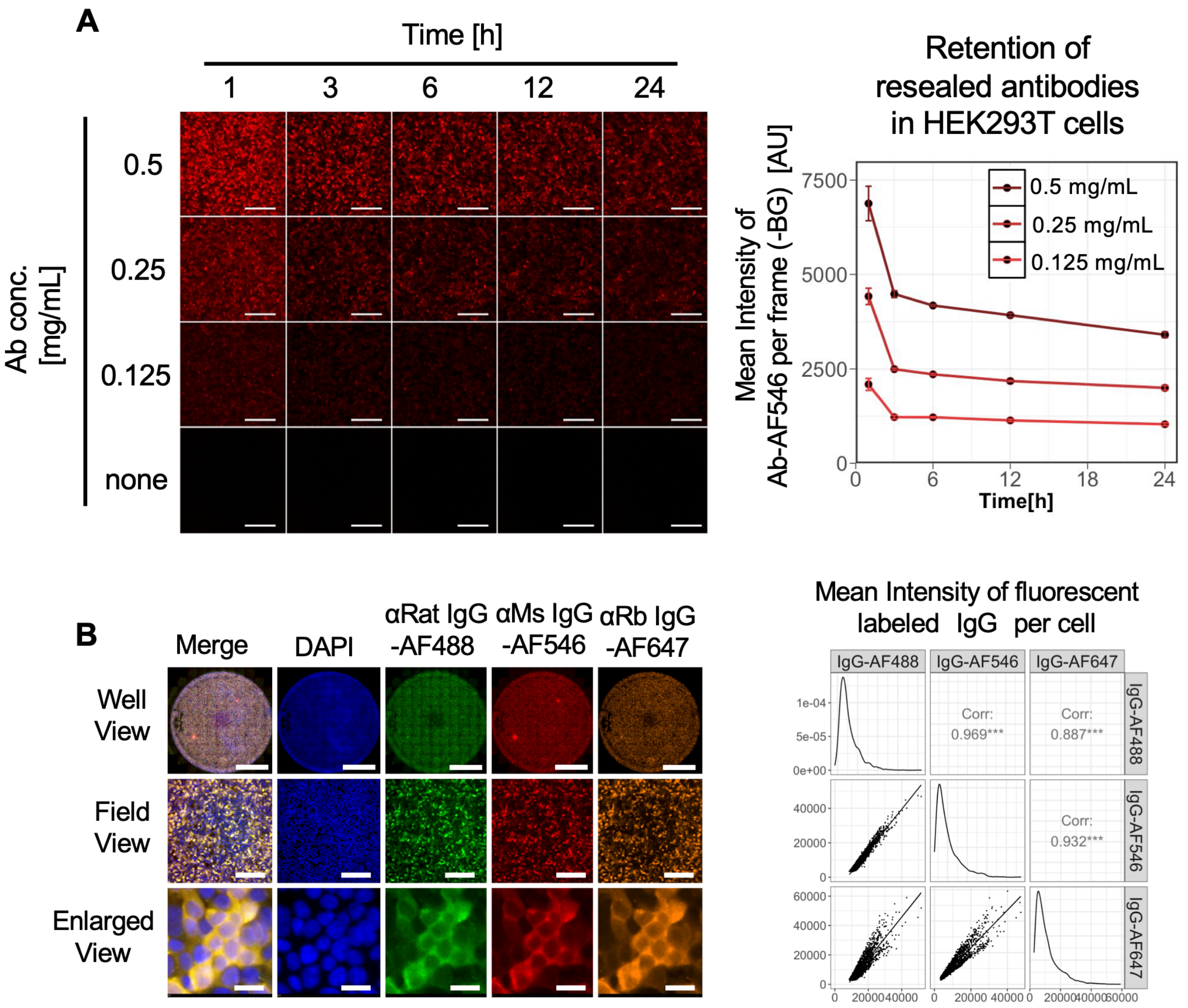

Fig. S3 Resealing experiments in which fluorescence-labeled antibodies were used.

(A) Evaluation of the retention of resealed antibodies. Microscopy images of HEK293T cells resealed with 0.500, 0.250, and 0.125 mg/mL of fluorescently-labeled antibodies; cells were imaged 1, 3, 6, 12, and 24 h after resealing. Scale bar: 200 µm. Line graph (right) shows the mean intensity of Alexa Fluor 546-labeled antibody, which was quantified per frame. The background fluorescence quantified from the 0 mg/mL antibody resealing condition was subtracted to obtain these results. The mean intensity of the fluorescently-labeled antibodies decreased immediately after resealing (~3 h), presumably because a fraction of highly fluorescent cells that were excessively permeabilized induced cell death and detached within the first 3 h. However, fluorescence intensity was well-retained across the

subsequent time course (3–24 h), with almost half of the antibodies being retained in the cells at 24 h after resealing. Data represent results from 15 frames (n = 15), expressed as mean ± SEM.

(B) Microscopy images of HEK293T cells simultaneously resealed with three different antibodies (0.2 mg/mL of each): anti-rat Alexa Fluor 488 goat IgG, anti-mouse Alexa Fluor 546 goat IgG, and anti-rabbit Alexa Fluor 647 goat IgG. Scale bar: 2,000 µm (Well view); 200 µm (Field view); 20 µm (Enlarged view). Pairwise plots of fluorescent intensities of the three different antibodies quantified per cell (right) showed high correlation between the three antibodies, indicating that although the permeabilization efficiency differed among cells, the ratio of the introduced antibodies was constant. Rb: rabbit, Ms: mouse.

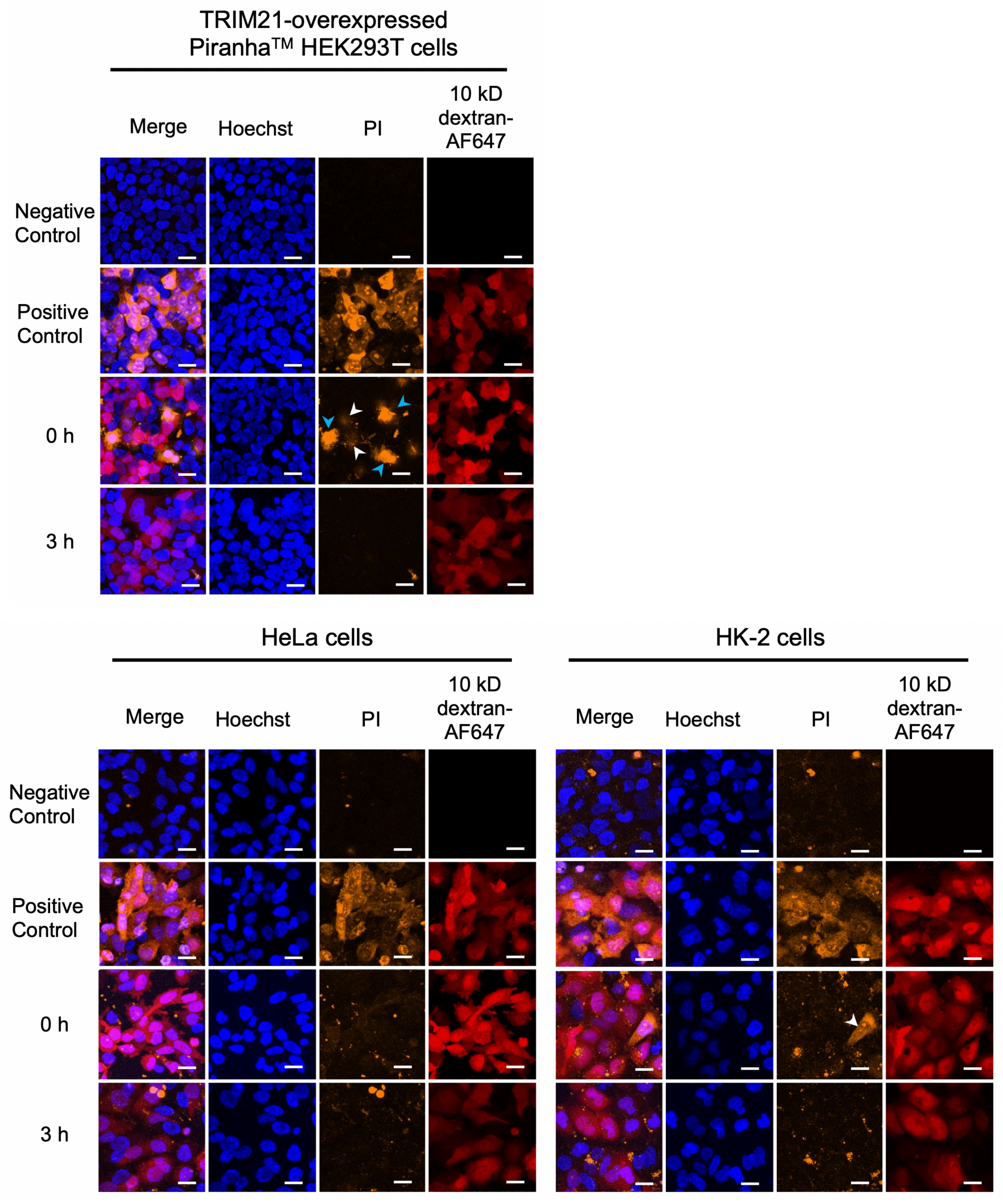

Fig. S4 Resealing efficiency in Piranha HEK293T, HeLa, and HK-2 cells.

To evaluate the membrane resealing efficiency, the cells were incubated with propidium iodide (PI) and Hoechst for 30 min immediately after the resealing step (0 h) or 3 h after resealing. Strong PI staining indicated dead cells (denoted by blue arrowheads), and weaker staining indicated cells with inefficient resealing (denoted by white arrowheads). Cells with inefficient resealing were rarely observed (approximately 1%) in the three cell lines, and most cells were PI-negative immediately after resealing. We could not accurately quantify the percentage of cells with incomplete staining as dead cells were strongly stained with PI, which may interfere with quantification. At 3 h after resealing, we did not detect any cells with inefficient resealing (PI-positive cells). For the negative control, intact cells were incubated with PI and Hoechst for 30 min. For the positive control, the cells were incubated with PI when streptolysin O pores were present (“semi-intact cells”) during the 5-min incubation step in TB buffer at 37°C. Dextran (10 kDa)-AF647 was used as the indicator of resealed cells. Scale bar: 20 μm.

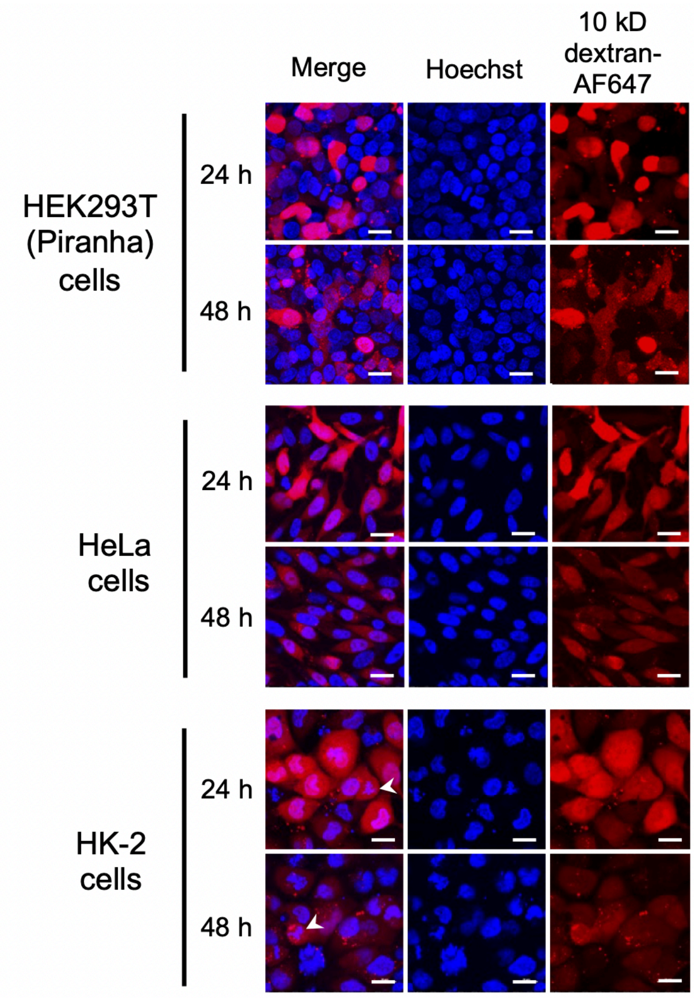

Fig. S5 Images of resealed cells incubated for 24 and 48 h.

HEK293T (Piranha), HeLa, and HK-2 cells were resealed with 10 kDa dextran-AF647 and incubated in the growth medium for 24 or 48 h. The images illustrate that resealed cells exhibited normal shapes and survived for at least 48 h. Resealed cells may retain the ability to proliferate, as indicated by the presence of resealed cells (dextran-positive cells) in the M phase (denoted by the white arrowhead). The laser settings were adjusted for each cell line and incubation time. Scale bar: 20 µm.

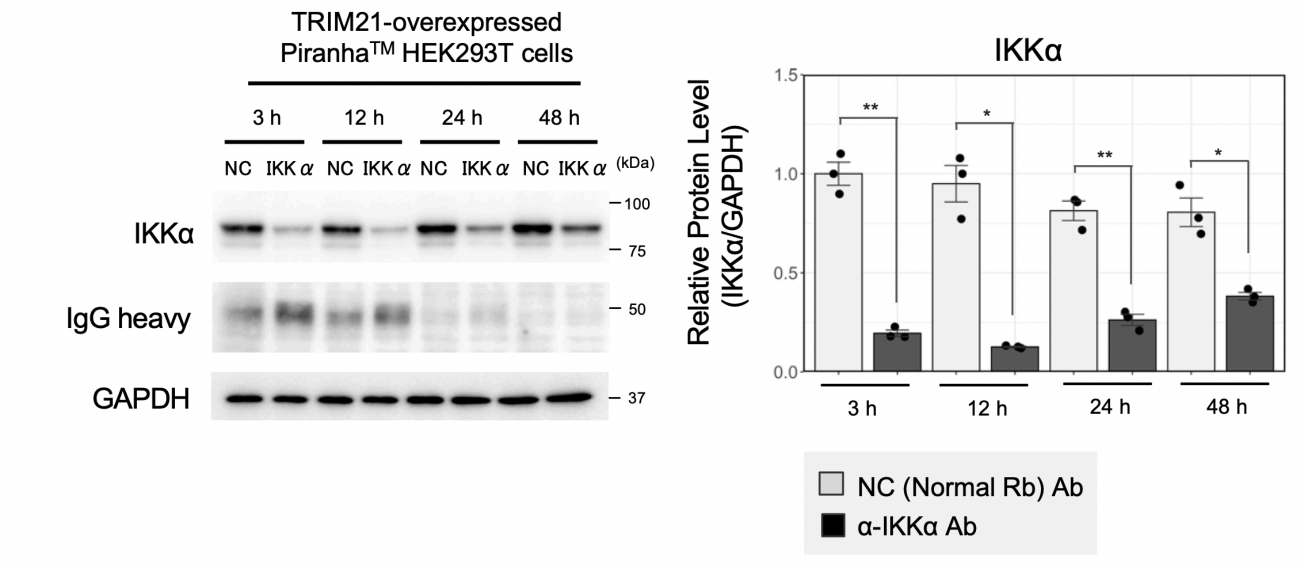

Fig. S6 Long-term protein knockdown efficiency in IKKα Trim-Away.

TRIM21-overexpressed Piranha HEK293T cells were subjected to IKKα Trim-Away by resealing with anti-IKKα antibodies, followed by incubation for 3, 12, 24, or 48 h, and cells were then collected for western blotting. IKKα knockdown efficiency was the highest at 12 h after resealing. IKKα levels recovered to some extent after 24 h of incubation, which occurred simultaneously with the decrease in resealed antibody levels. Normal rabbit antibody levels also exhibited a time-dependent decrease, indicating that the resealed antibodies were degraded regardless of their target-binding ability. This result was different from the findings in Fig. S3A, in which fluorescently labeled antibodies were retained at least 24 h after resealing. This difference may be attributable to fluorescence labeling, difference in the antibody itself, or difference in the evaluation method. NC: negative control, Rb: rabbit. Data expressed as mean ± SEM (n = 3). *P < 0.05, **P < 0.01.

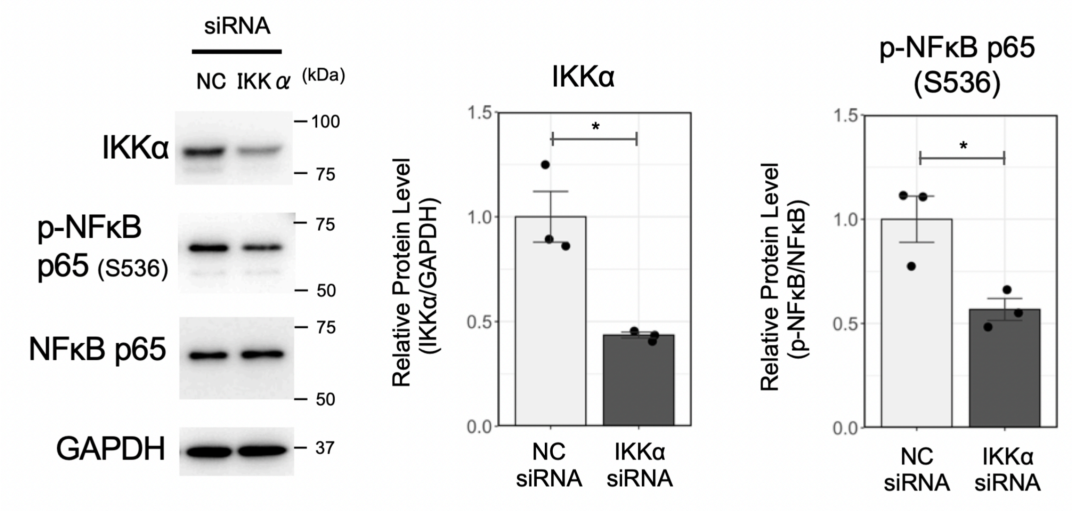

Fig. S7 IKKα siRNA knockdown led to reduced NF-κB p65 phosphorylation.

Piranha HEK293T cells were transfected with IKKα siRNA and incubated for 24 h. IKKα knockdown resulted in a 56% reduction in IKKα levels and 43% reduction in NF-κB p65 phosphorylation. NC: negative control. Data are expressed as mean ± SEM (n = 3). *$P < 0.05$.

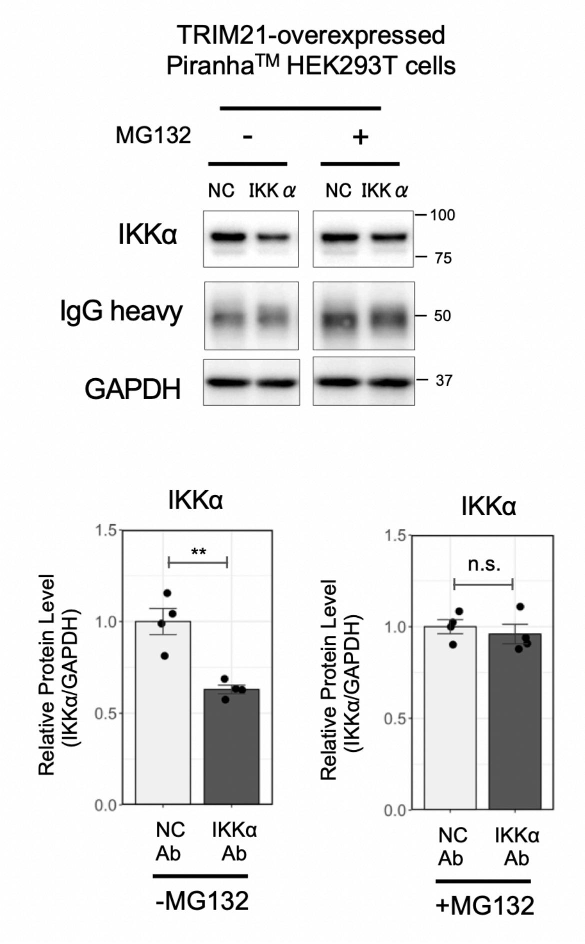

Fig. S8 MG132 treatment inhibited Trim-Away–mediated target protein degradation.

Proteasomal degradation was inhibited by treating Piranha HEK293T cells with MG132. The cells were pretreated with 25 µM MG132 for 1–3 h and then subjected to resealing procedures. MG132 was supplemented during the resealing steps and during incubation after resealing. The cells were collected 1–3 h after resealing for western blotting. Data are expressed as mean ± SEM (n = 4). **P < 0.01, ns: not significant. NC: negative control.

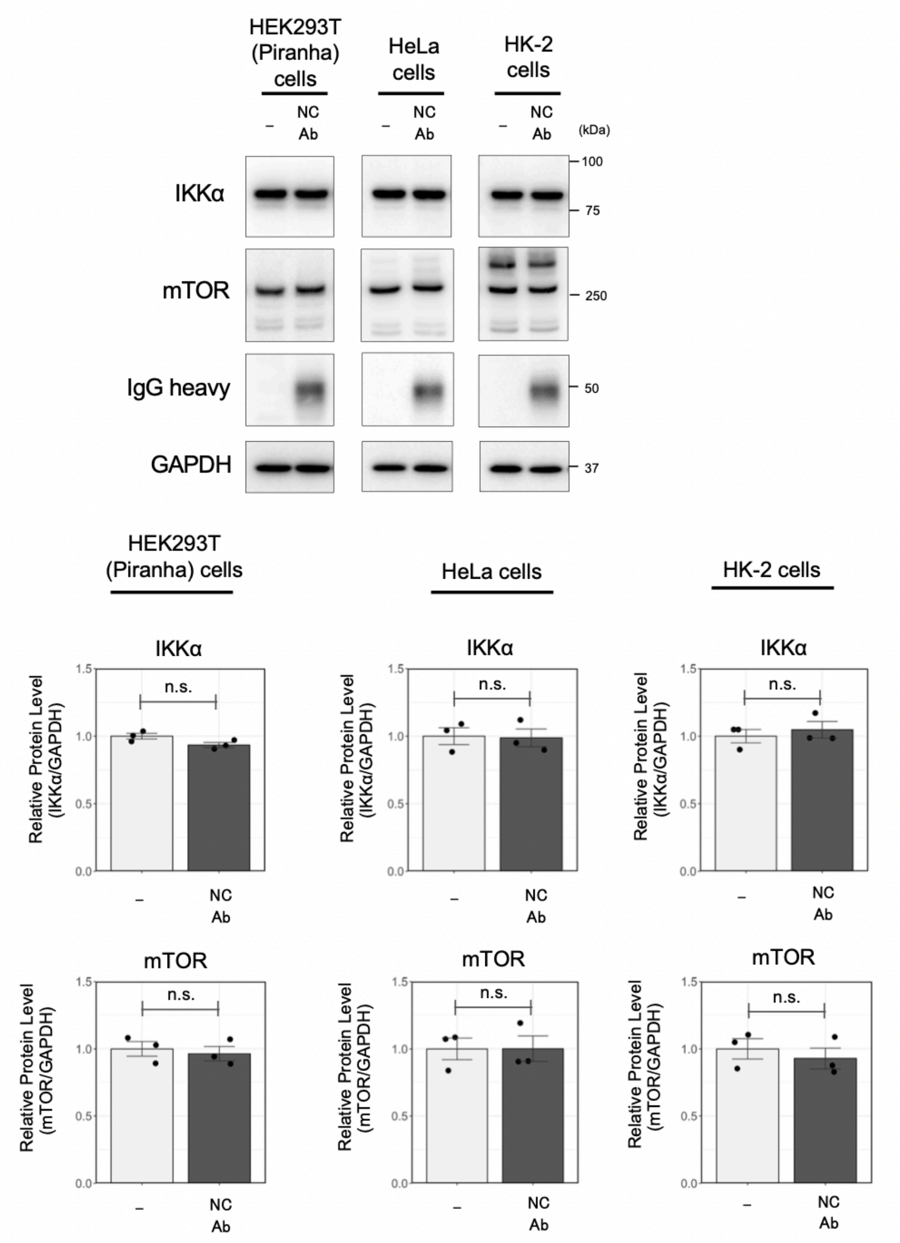

Fig. S9 Resealing of negative control antibody does not alter the levels of IKKα and mTOR.

Piranha HEK293T, HeLa, and HK-2 cells were resealed with 0.5 mg/mL normal rabbit antibodies and collected 3 h after resealing for western blotting. For mTOR blots of HK-2 cell lysates, an extra band was observed, which likely represented mTOR that underwent glycosylation or another post-translational modification. Data are expressed as mean ± SEM (n = 3). ns: not significant. NC: negative control.

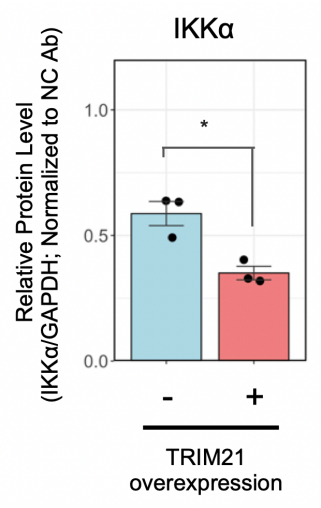

Fig. S10 TRIM21-overexpressing cells exhibited higher degradation efficiency of IKKα (related to Fig. 2A and C).

HEK293T or TRIM21-overexpressing Piranha HEK293T cells were incubated for 3 h after resealing with 0.25 mg/mL anti-IKKα antibodies or the same level of normal rabbit antibody as a nontargeting negative control (NC). The degradation efficiency of IKKα was normalized to each NC, and the difference between the cells was compared. TRIM21-overexpressing Piranha HEK293T cells exhibited higher IKKα degradation efficiency than normal HEK293T cells. Data are expressed as mean ± SEM (n = 3). **P < 0.01. NC: negative control.

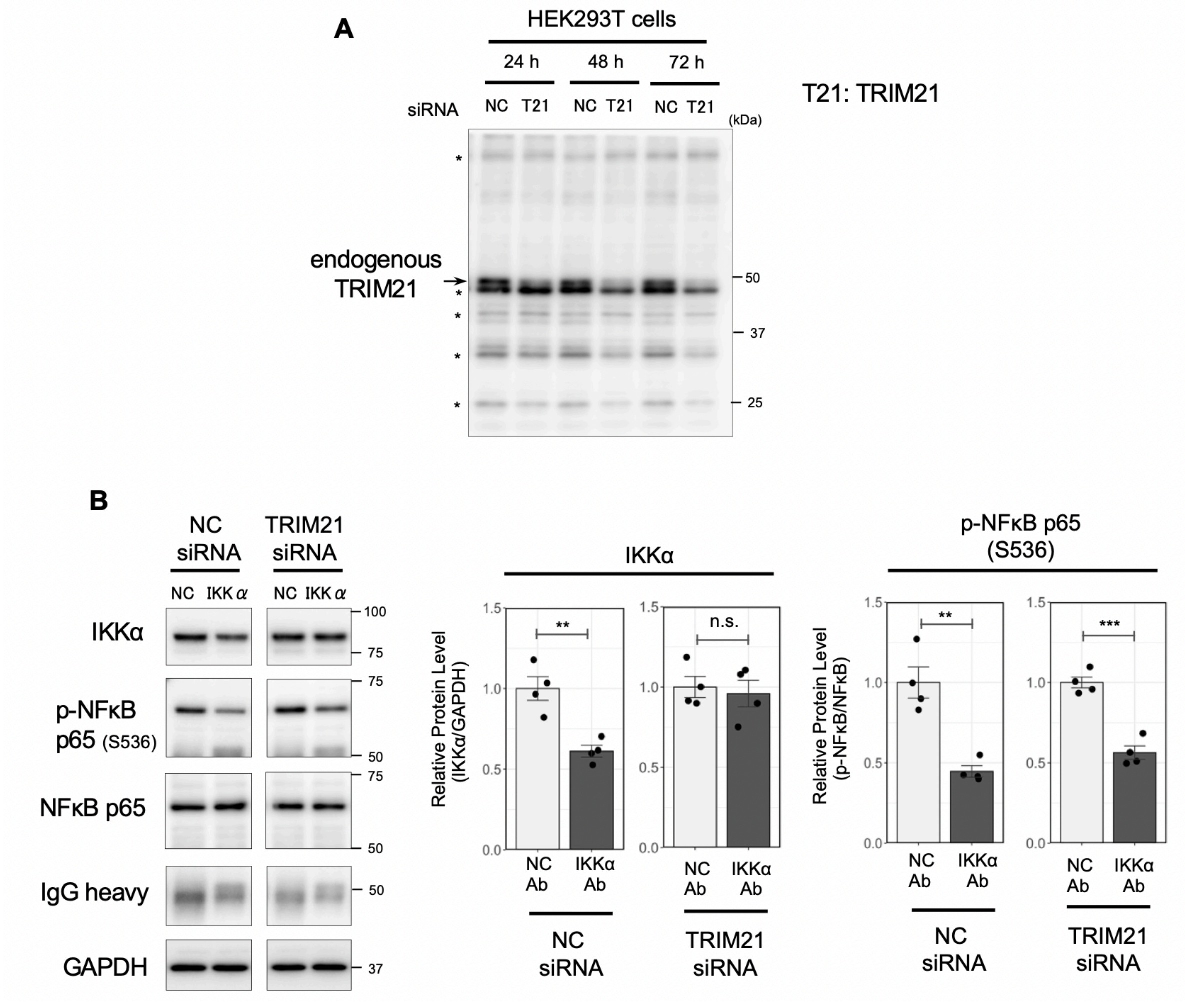

Fig. S11 Trim-Away of IKKα in HEK293T cells with TRIM21 knockdown (KD).

(A) Efficiency of siRNA-mediated TRIM21 KD was tested in HEK293T cells. Cells were collected at 24, 48, or 72 h after transfection and assessed via western blotting. The estimated endogenous TRIM21 band is indicated by the black arrow, and extra bands are indicated by asterisks. T21: TRIM21.

(B) HEK293T cells were transfected with TRIM21 siRNA or negative control siRNA, incubated for 48 h, and subjected to IKKα Trim-Away. Antibody delivery via cell resealing was performed using the standard resealing procedure except that the cytosol used in the resealing step was obtained from TRIM21 KD HEK293T cells. The cells were collected 3 h after resealing for western blotting. The degradation efficiency of IKKα was normalized to each negative control (NC Ab), and the difference between TRIM21 KD and negative control cells was analyzed. IKKα degradation was inhibited in TRIM21 KD cells compared with negative control cells (NC siRNA). Data are expressed as mean ± SEM (n = 4). **P < 0.01, ***P < 0.001, ns: not significant. NC: negative control.

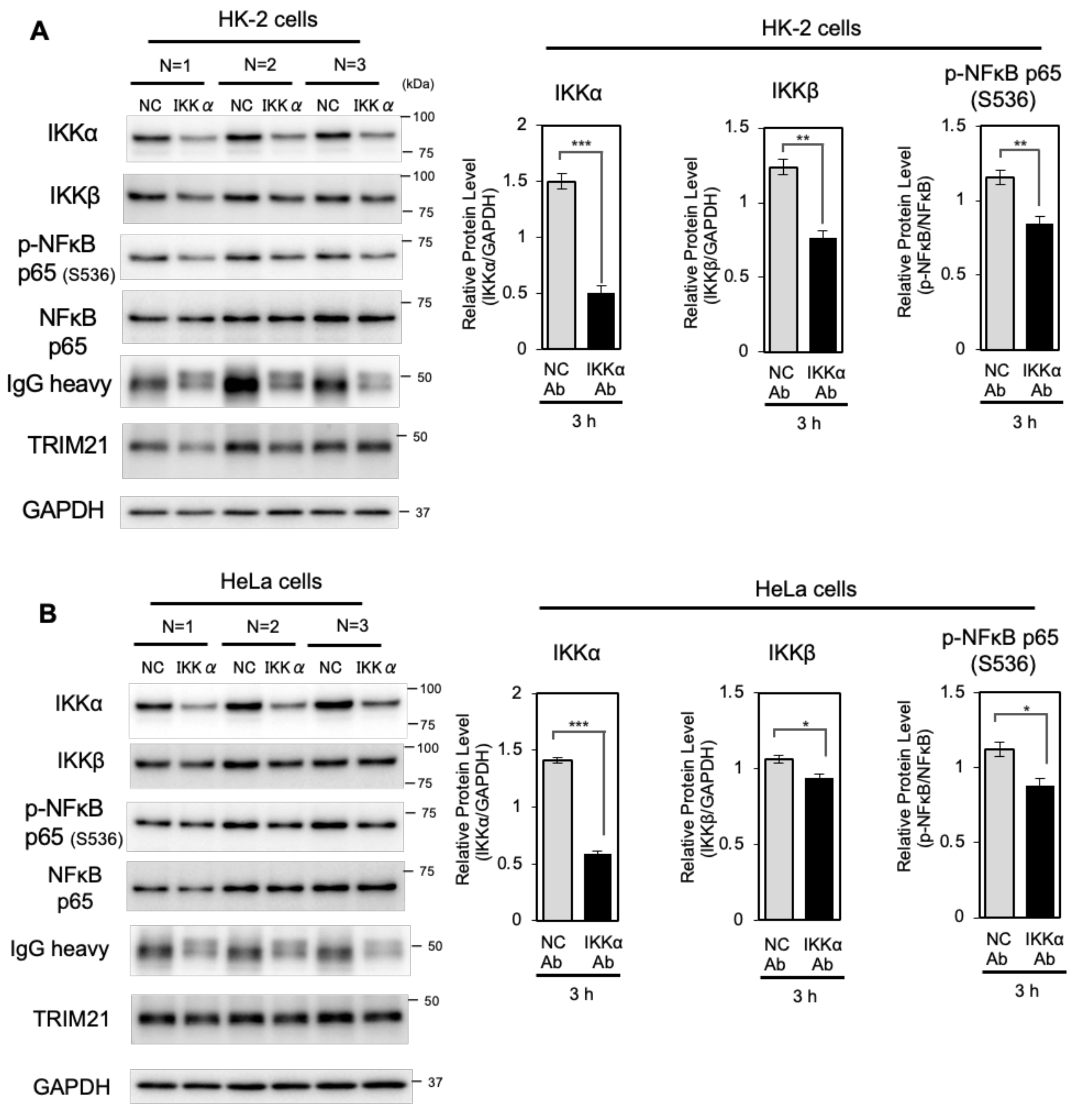

Fig. S12 Cell resealing-mediated Trim-Away of IKKα in HK-2 and HeLa cells.

Western blotting of HK-2 and HeLa cells incubated for 3 h after resealing with 0.25 mg/mL of anti-IKKα antibody (clone Y463, abcam, Cat# ab169743) or the same amount of normal rabbit antibody as a negative control (NC).

(A) In HK-2 cells, resealing of anti-IKKα antibody led to a 67% reduction in IKKα protein level; the IKKβ protein level and phosphorylated NF-κB p65 level also significantly decreased. Data expressed as mean ± SEM (n = 3). $**P < 0.01$, $***P < 0.001$.

(B) In HeLa cells, resealing of anti-IKKα antibody (clone Y463, abcam, Cat# ab169743) led to a 58% reduction in IKKα protein level; IKKβ protein level slightly decreased. Phosphorylation of NF-κB p65 was reduced under anti-IKKα-resealed conditions. Data expressed as mean ± SEM (n = 3). $*P < 0.05$, $***P < 0.001$.

A

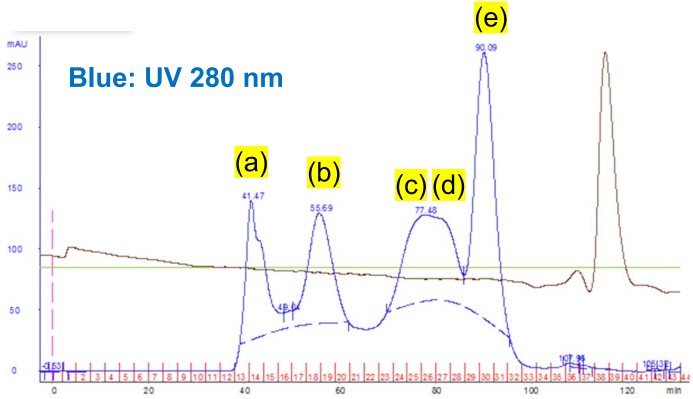

B

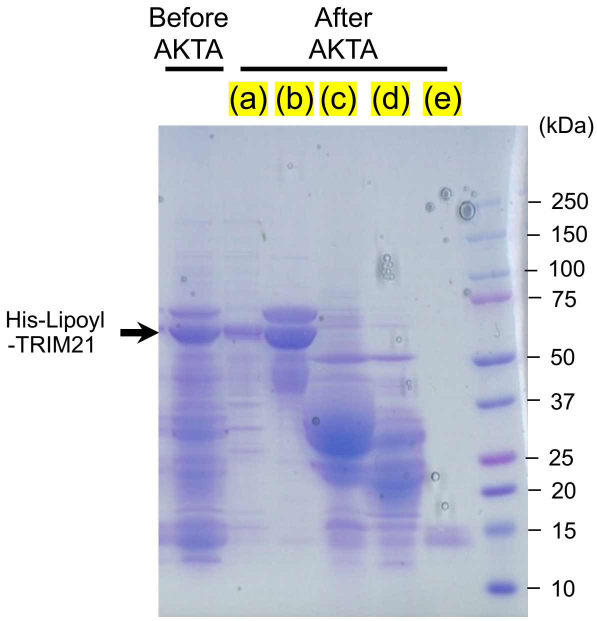

Fig. S13 Purification of His-Lipoyl-TRIM21 protein.

(A) Chromatogram from an automated run of His-Lipoyl-TRIM21 protein purification using the HiLoad 16/600 Superdex 200 PG size exclusion column and the AKTA pure purification system. Peak fractions (a–e) were pooled and concentrated. The blue line shows UV 280 nm.

(B) Purification of the His-Lipoyl-hTRIM21 protein was analyzed by Coomassie Brilliant Blue staining. The His-Lipoyl-hTRIM21 protein was eluted in peak fraction (b).

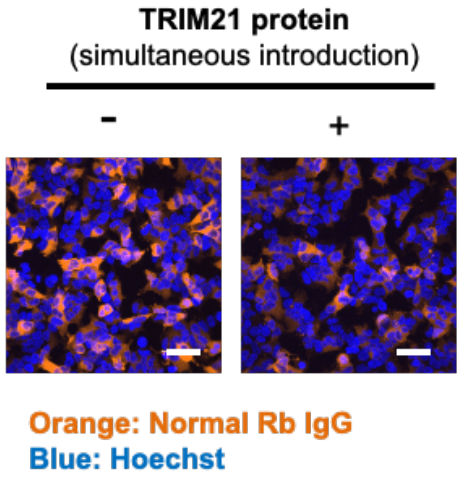

Fig. S14 Simultaneous introduction of antibody and TRIM21 protein results in decreased delivering efficiency of the antibody.

HEK293T cells were resealed with normal rabbit IgG, after which they were incubated for 1 h and fixed for analysis. Compared to the cells resealed with antibody alone (left panel), cells resealed simultaneously with antibody and TRIM21 protein showed a decreased amount of introduced antibody. Scale bar: 50 µm. Rb: rabbit.

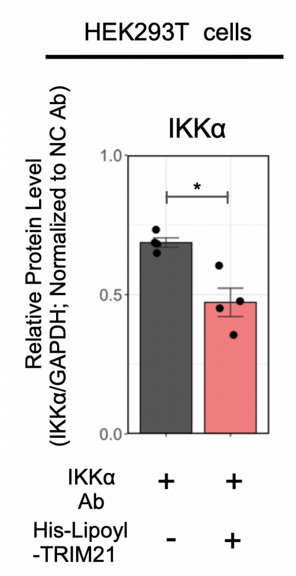

Fig. S15 Enhanced degradation of IKKα via supplementation of exogenous TRIM21 (related to Fig. 3C).

Western blotting bands presented in Fig. 3C were quantified, and the degradation efficiency was compared between conditions with or without exogenous TRIM21 ("His-Lipoyl-TRIM21"). Anti-IKKα antibody-resealed conditions were normalized to those of their respective negative controls (conditions resealed with normal rabbit antibody). Data are expressed as mean ± SEM (n = 4). *P < 0.05.

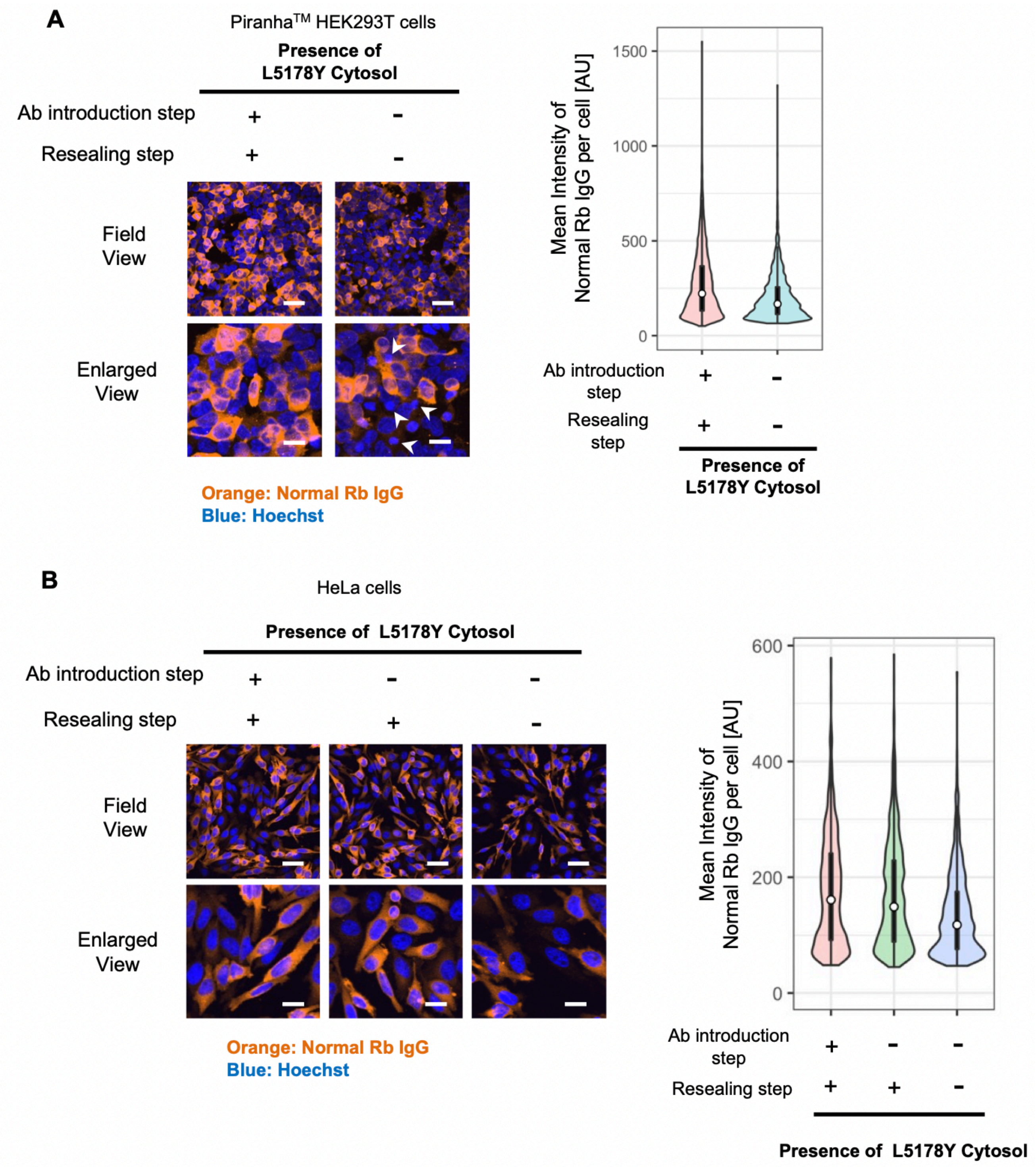

Fig. S16 The presence of cytosol in the resealing step enhances resealing efficiency.

(A) Piranha™ HEK293T cells were resealed with normal rabbit IgG in the presence or absence of L5178Y cytosol in the antibody introduction step and the resealing step. Cells resealed in the presence of L5178Y cytosol showed an enhanced retention rate of the introduced antibody (left panel) compared

to the cells resealed in the absence of cytosol (right panel). Cells resealed in the absence of cytosol showed higher susceptibility to cell death as indicated by decreased cell number and nuclear shrinkage (white arrowhead), likely due to inefficient resealing. The mean intensity was quantified per cell (n = 2945, 2525). Scale bar: 50 μm. Rb: rabbit.

(B) HeLa cells were resealed with normal rabbit IgG in the presence or absence of L5178Y cytosol in the antibody introduction step and/or the resealing step. The presence of cytosol during the resealing step enhanced the retention rate of the introduced antibody (comparing the right and middle panels), whereas the presence of cytosol during the antibody introduction step did not further enhance the retention rate of the introduced antibody (comparing the middle and left panels). Scale bar: 50 μm. The mean intensity per cell was quantified (n = 808, 997, 740). Scale bar: 50 μm. Rb: rabbit.

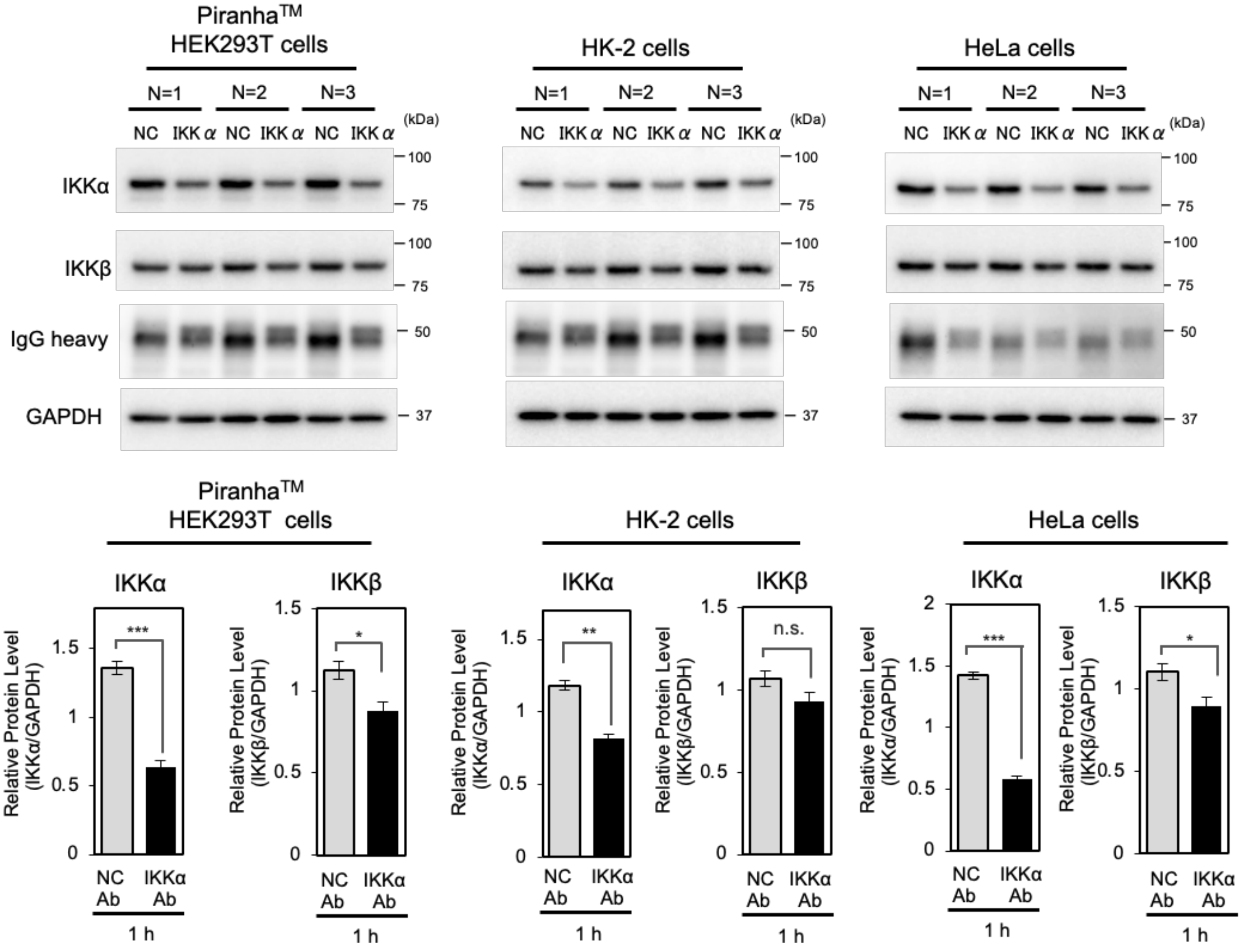

Fig. S17 Cell resealing-mediated Trim-Away degradation of IKKα occurs without supplementation of exogenous cytosol.

TRIM21-overexpressed Piranha^{TM} HEK293T cells, HK-2 cells, and HeLa cells were resealed with anti-IKKα antibody (clone Y463, Abcam, Cat# ab169743) without the supplementation of exogenous cytosol throughout the resealing procedure. The cells were incubated for 1 h and collected for western blotting analysis. Resealing of anti-IKKα antibody resulted in 53%, 31%, 59% reduction of IKKα in Piranha^{TM} HEK293T cells, HK-2 cells, and HeLa cells, respectively. Data are expressed as mean ± SEM (n = 3). *P < 0.05, **P < 0.01, ***P < 0.001, ns: not significant. NC: negative control.

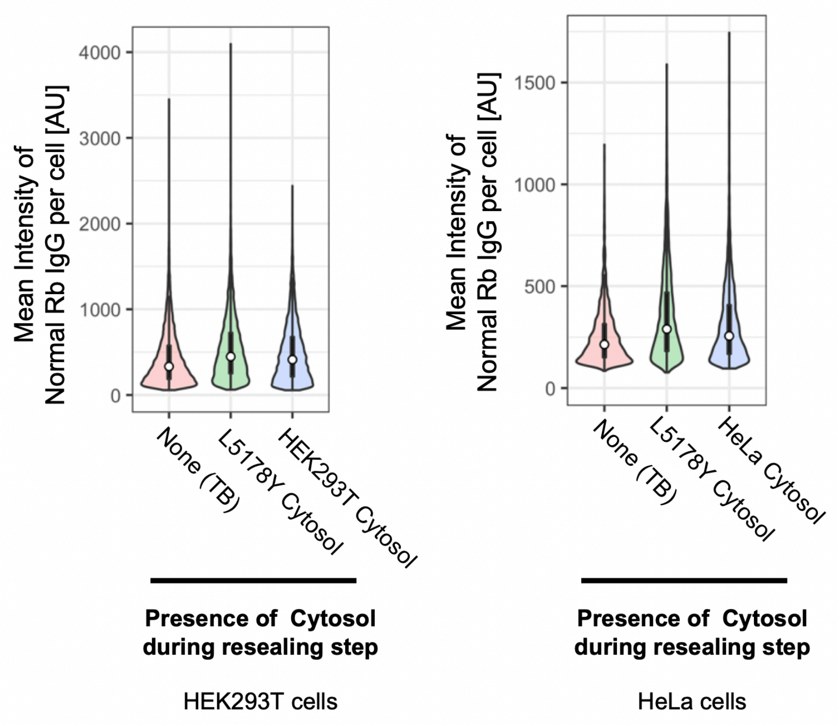

Fig. S18 Comparison of the antibody resealing efficiency (Related to Fig. 3D).

Comparison of the antibody resealing efficiency between cells that were incubated without cytosol (TB), with L5178Y cytosol, or with cytosol obtained by the new freeze-thaw protocol, during the resealing step. HEK293T cells or HeLa cells were resealed with normal rabbit IgG, after which they were incubated for 1 h and fixed for analysis. The images shown in Fig.3D were quantified per cell (n = 10138, 10445, 10538 for HEK293T cells, n=2322, 2888, 2638 for HeLa cells).

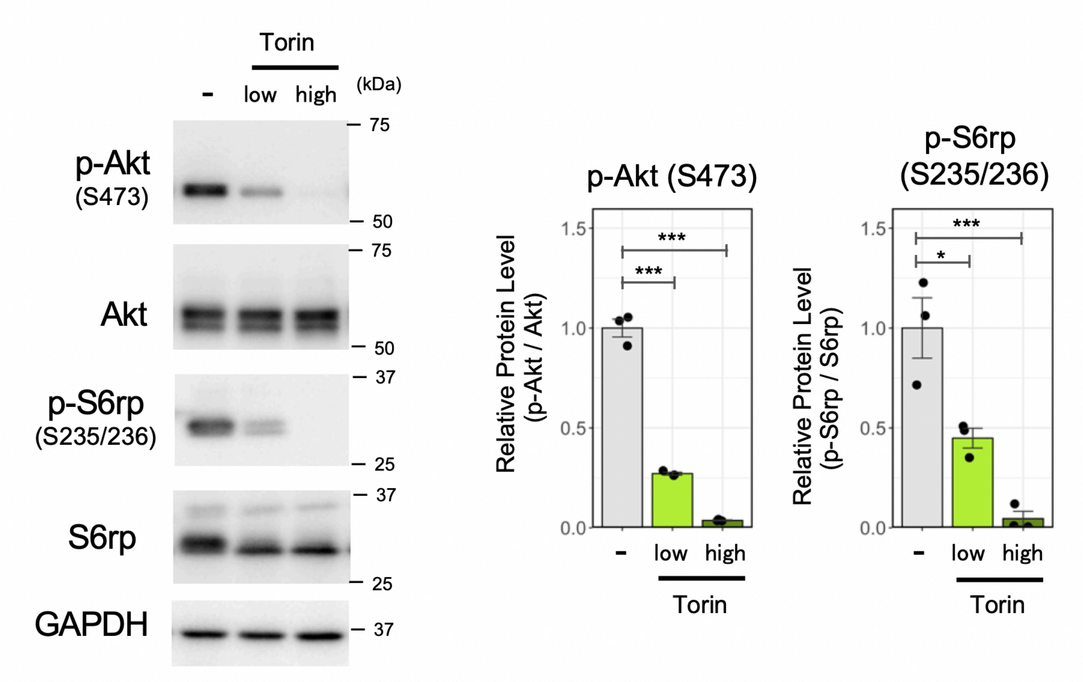

Fig. S19 mTOR inhibition by Torin led to decreased phosphorylation of Akt and S6rp.

Piranha HEK293T cells were treated with 25 (“low”) or 250 nM (“high”) Torin for 3 h and collected for western blotting. Data are expressed as mean ± SEM (n = 3). *P < 0.05, ***P < 0.001.

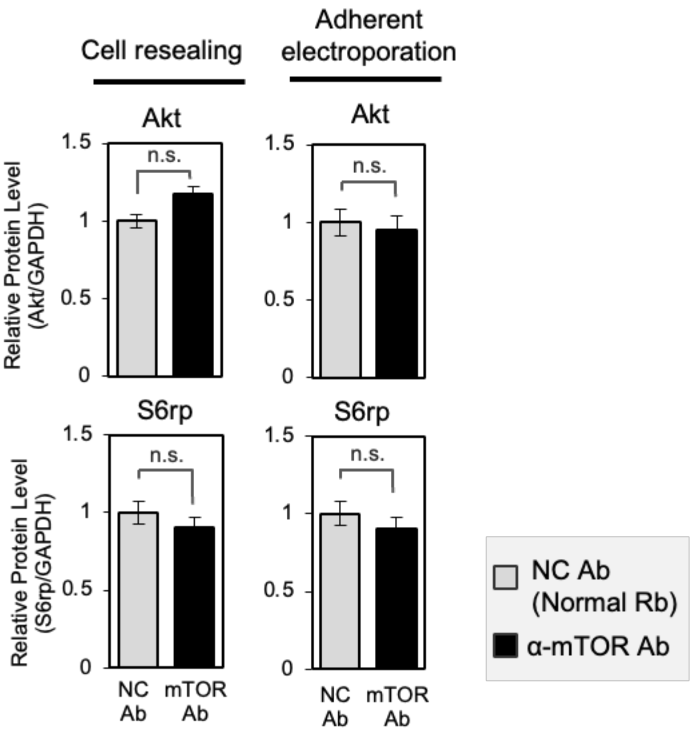

Fig. S20 Trim-Away–mediated degradation of mTOR using the cell resealing technique or adherent electroporation (related to Fig. 5B).

The bar graph presents the quantification of the bands in Fig. 5B. The total levels of Akt and S6rp were not altered in the mTOR Trim-Away experiment. Data are expressed as mean ± SEM (n = 3). ns: not significant. Rb: rabbit, Ab: antibody.

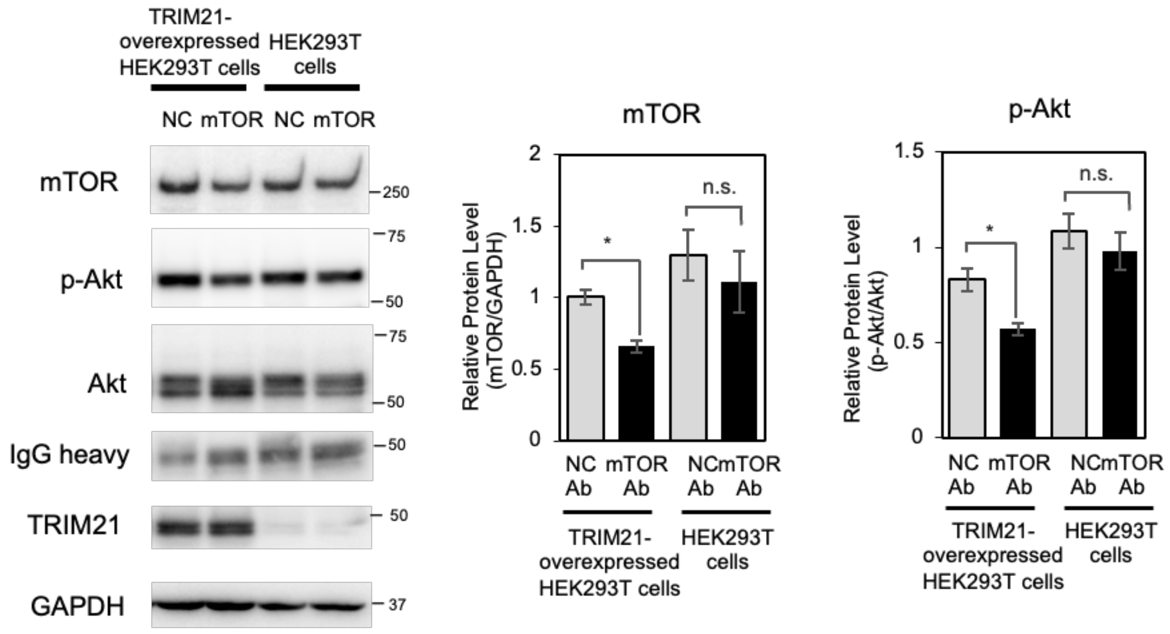

Fig. S21 Additional expression of exogenous TRIM21 is required for efficient degradation of mTOR.

The degradation efficiency of mTOR in TRIM21-overexpressed Piranha^{TM} HEK293T cells was compared with that in normal HEK293T cells. Cells were resealed with 0.5 mg/mL of anti-mTOR antibody (clone 7C10, CST, Cat#2983BF) or the same amount of normal rabbit antibody as a negative control (NC), after which they were incubated for 12 h and collected for western blotting analysis. Endogenous TRIM21 was not sufficient to degrade a significant amount of mTOR in HEK293T cells, whereas cells with additional expression of exogenous TRIM21 showed significant mTOR degradation. Data expressed as mean ± SEM (n = 3). *P < 0.05, ns: not significant. NC: negative control.

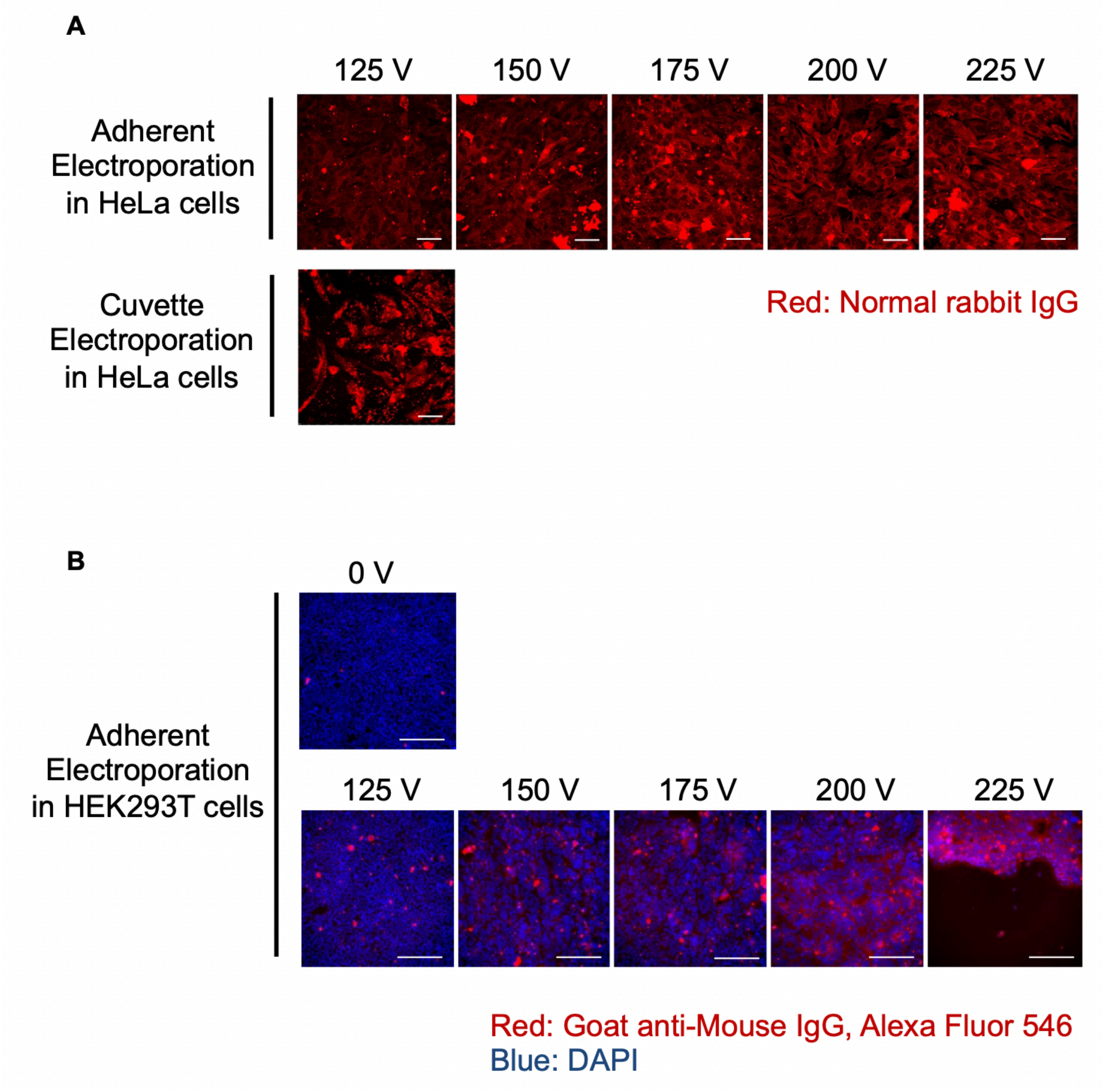

Fig. S22 Optimal pulse condition for electroporation-mediated antibody delivery.

(A) Microscopy images of HeLa cells into which normal rabbit IgG had been delivered by electroporation and, 20 h later, cells had been fixed for immunofluorescence labeling of the introduced antibodies. For adherent electroporation, HeLa cells were grown on 96-well plates and cell-culture-plate electrodes were applied. The poring pulses were tested at 125–225 V, and the antibody was efficiently delivered with a pulse of 175–200 V. Cuvette electroporation with a 150 V poring pulse (lower left panel) resulted in inefficient cytosolic antibody delivery, with most antibodies found stuck to the cell surface. Scale bar: 50 µm.

(B) Microscopy images of HEK293T cells into which Alexa Fluor 546 labeled goat anti-mouse IgG had been delivered by electroporation and, 3 h later, cells had been fixed. The antibody was efficiently delivered with a pulse of 200 V. Scale bar: 200 µm.

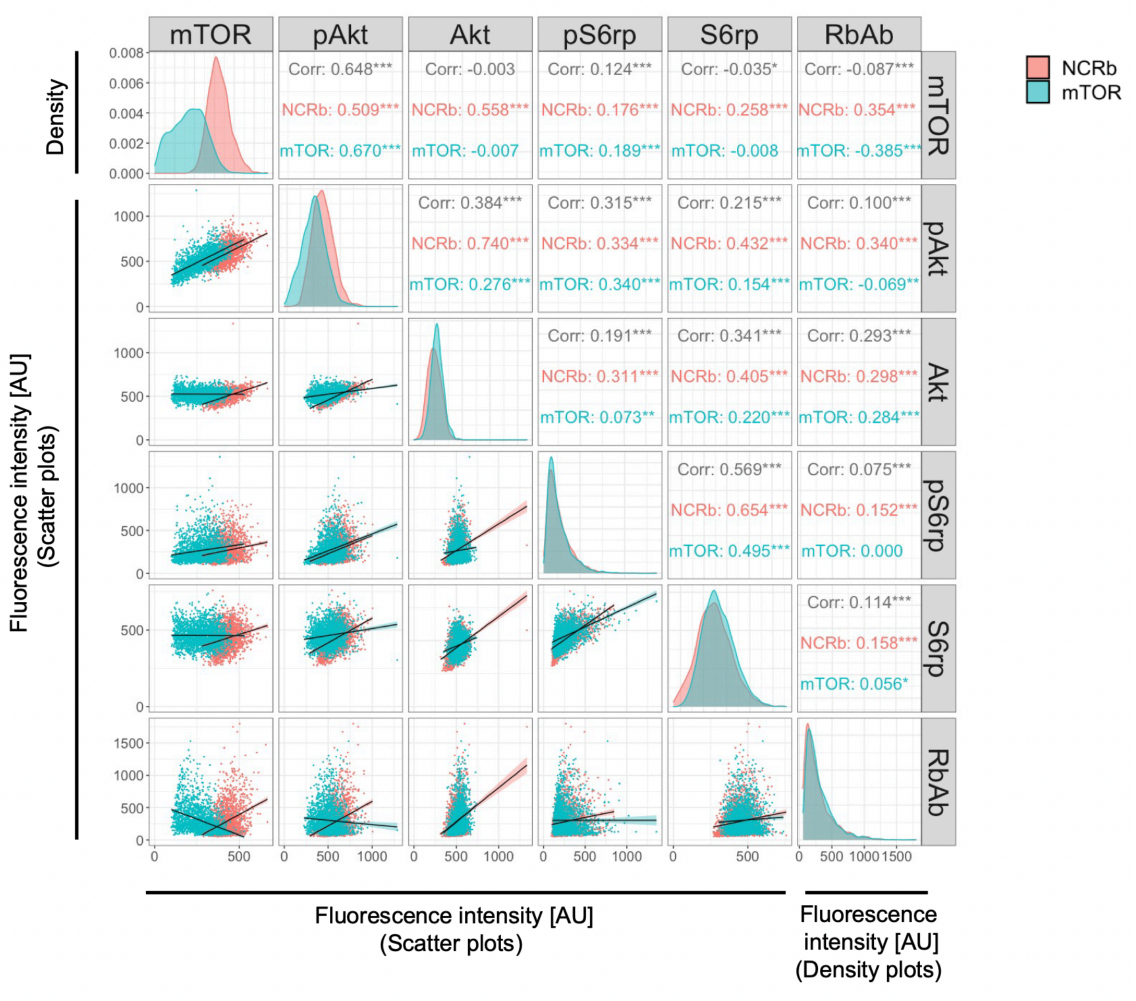

Fig. S23 Pairwise plots of multivariate data obtained from the cell resealing-mediated mTOR Trim-Away experiment.

Single-cell multidimensional data containing the mean intensities of the stained proteins [obtained from the mTOR Trim-Away experiment (see Fig. 6B)] were plotted. The upper panels show correlations between variables (Corr: correlation of both NCRb and mTOR conditions combined, NCRb: correlation of negative control condition, mTOR: correlation of anti-mTOR antibody resealing condition), the lower panels show scatter plots of the variables, and the diagonal shows the density plots of the variables. Histograms of fluorescence intensity indicate that the anti-mTOR antibody resealing condition (color-coded pink) had lower mTOR and pAkt intensities compared with those of the negative control (color-coded blue). Scatterplots showing the fluorescence of resealed anti-mTOR antibodies vs. immunofluorescence-stained mTOR fluorescence indicate a weak negative correlation between the variables. Similarly, a weak negative correlation existed between pAkt and the resealed anti-mTOR antibody. RbAb: rabbit antibody (Normal rabbit IgG or anti-mTOR antibody), NC: negative control, Rb: rabbit, Corr: correlation. $^*P < 0.05$, $^{**}P < 0.01$, $^{***}P < 0.001$.

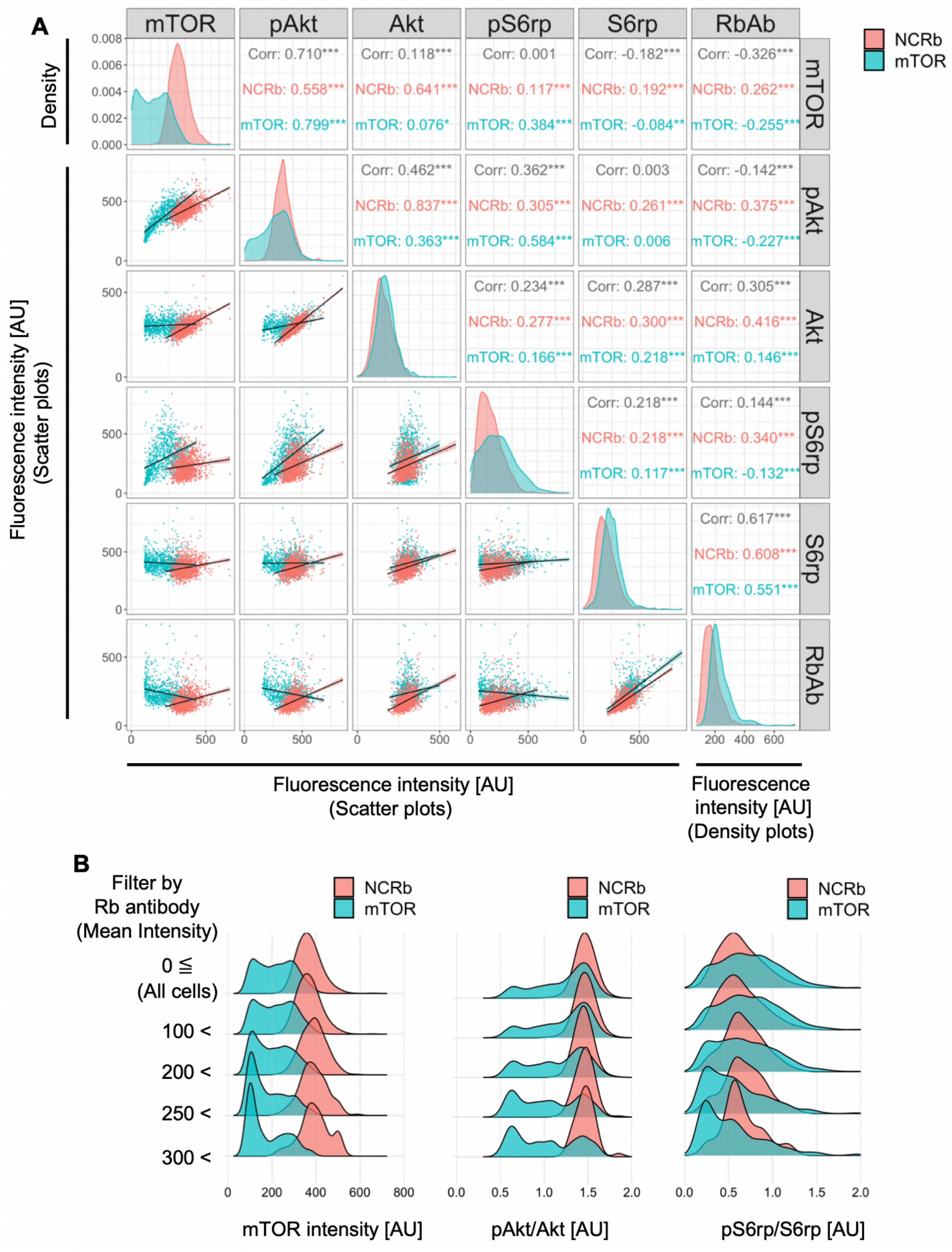

Fig. S24 Single-cell analysis of adherent electroporation-mediated Trim-Away targeting mTOR.

The cyclic immunofluorescence analysis framework described in Fig. 6A was applied for single-cell analysis of adherent electroporation-mediated Trim-Away targeting mTOR. Anti-mTOR antibodies (clone 7C10, CST, Cat#2983BF) were introduced into TRIM21-overexpressed Piranha^{TM} HEK293T cells grown in 96-well plates using adherent electroporation; the cells were then incubated for 12 h and fixed for cyclic immunofluorescence. Images were acquired from the well center to obtain images of well-electroporated cells. After repeated rounds of immunofluorescence staining, imaging, and bleaching, the resultant images were aligned and analyzed. RbAb: rabbit antibody (Normal rabbit IgG or anti-mTOR antibody), NC: negative control, Rb: rabbit.

(A) Pairwise plots of multivariate data obtained from the adherent electroporation-mediated mTOR Trim-Away experiment. The upper panels show correlations between variables (Corr: correlation of both NCRb and mTOR conditions combined, NCRb: correlation of negative control condition, mTOR: correlation of anti-mTOR antibody resealing condition), the lower panels show scatter plots of the variables, and the diagonal shows the density plots of the variables. Histograms of fluorescence intensities indicate that anti-mTOR antibody-electroporated conditions (color-coded pink) had lower mTOR and pAkt intensities compared with those in the negative control (color-coded blue). Scatterplots showing the fluorescence of anti-mTOR antibody vs. immunofluorescence-stained mTOR fluorescence show a weak negative correlation between the variables. A weak negative correlation was also detected between pAkt and the introduced anti-mTOR antibody. Corr: correlation. $*P < 0.05$, $**P < 0.01$, $***P < 0.001$.

(B) Density plots showing mTOR intensity, pAkt/Akt, and pS6rp/S6rp of cells electroporated with mTOR or normal rabbit IgG (NCRb). The mean intensities of the phosphorylated forms were normalized against the total level of the respective proteins, represented as pAkt/Akt and pS6/S6rp. Single-cell data were filtered by the mean fluorescence intensity of the introduced antibody. Differences in the distribution between anti-mTOR antibody-electroporated cells and the negative control became more pronounced as the data were narrowed down to the most efficiently electroporated cells; thus, mTOR was efficiently degraded and the downstream targets were inactivated by the introduction of anti-mTOR antibody. AU: arbitrary unit.

Table S1

Comparison between cell resealing to other antibody introduction methods for Trim-Away

	Cell resealing	Adherent electroporation	Capillary electroporation	Microinjection	Carrier-mediated
Adherent/Suspension	Adherent	Adherent	Suspension-only	Adherent	Adherent
Throughput	High	Mid	Mid	Low	High
Scalability	High	Low	Mid	Low	High
Rapid delivery	Rapid	Rapid	Rapid	Rapid	Slow
In vivo translation	Not amenable	Not amenable	Not amenable	Amenable	Amenable
Assay	WB/Microscopic	Microscopic-only	WB/Microscopic	WB/Microscopic	WB/Microscopic

2. Supplemental Methods

2.1. Reagents or resource

REAGENT or RESOURCE	SOURCE	IDENTIFIER
Antibodies		
Akt (clone C67E7)	Cell Signaling Technology	Cat#4692S; RRID: AB_915783
Akt(pSer473) (clone D9E)	Cell Signaling Technology	Cat#4060P; RRID:AB_2315049
GAPDH	Millipore	Cat#MAB374
mTOR (clone 7C10)	Cell Signaling Technology	Cat#2983; RRID:AB_2105622
mTOR(pSer2442) (clone D9C2)	Cell Signaling Technology	Cat#5536; RRID:AB_10691552
S6RP (clone 54D2)	Cell Signaling Technology	Cat#2317; RRID:AB_2238583
S6RP(pSer235_236) (clone D57.2.2E)	Cell Signaling Technology	Cat#4858; RRID:AB_916156
Anti-Mouse IgG (H+L), HRP Conjugate	Promega	Cat#W4021
Alexa-488-anti-mouse IgG	molecular probes	Cat#A11029

Alexa-546-anti-mouse IgG	molecular probes	Cat#A11030
Alexa-647-Anti-mouse IgG	molecular probes	Cat#A21236
Alexa-488-anti-rabbit IgG	molecular probes	Cat#A11034
Alexa-546-anti-rabbit IgG	molecular probes	Cat#A11035
Alexa-647-Anti-rabbit IgG	molecular probes	Cat#A21245
IKKα (clone Y463)	abcam	Cat#ab169743
IKKα+IKKβ (clone EPR16628)	abcam	Cat#ab240210
IKKβ (clone EPR6043)	abcam	Cat#ab171364
IKKα (clone 14A231)	Novus Biologicals	Cat#NB100-56704
IKKα	Arigo Biolaboratories	Cat#ARG54650
NFkB(pSer536) (clone 93H1)	Cell Signaling Technology	Cat#3033
NFkB (clone D14E12)	Cell Signaling Technology	Cat#8242
IKKα	Cell Signaling Technology	Cat#2682
IKKβ (clone 2C8)	Cell Signaling Technology	Cat#2370
LC3 (clone 4E12)	MBL	Cat#M152-3
GFP	MBL	Cat#598
Normal Rabbit IgG, Whole Molecule, Purified	Fujifilm	Cat#148-09551

Normal Mouse IgG, Whole Molecule, Purified	Fujifilm	Cat#140-09511
Ro/SSA (clone D-12)	Santa Cruz	Cat#sc-25351
mTOR (clone 7C10) in PBS	CST	Cat#2983BF
Other regents		
Stripping Solution	WAKO	Cat#193-16375
Hoechst 33342	Dojindo	Cat#H346-07951
Precision Plus Protein Dual Color Standards	Bio-Rad	Cat#161-0394
Adenosine triphosphate (ATP)	Sigma-Aldrich	Cat#161-0394
Creatine phosphate	Roche	Cat#10621714001
Creatine kinase	Roche	Cat#10 127566
Guanosine triphosphate (GTP)	Takara	Cat#4042
Streptolysin O (SLO)	Bioacademia	Cat#01-531
MG132	Fujifilm	Cat#139-18451
Torin	Tocris	Cat#4247
Propidium iodide	molecular probes	Cat#3566
DAPI	DOJINDO	Cat#342-07431

Triton-X 100	WAKO	Cat#04605-250
Bovine Serum Albumin	EQUITECH-BIO	Cat#BAH-0500
Can Get Signal	TOYOBO	Cat#NKB-101
Western Lightning Chemiluminescence Reagent PLUS-ECL	PerkinElmer	Cat#NEL104001EA
Penicillin Streptomycin 10,000U/ml	Gibco	Cat#15140122
2.5% Trypsin(10x)	Gibco	Cat#15090046
Stripping Solution	WAKO	Cat#193-16375
GENETICIN	Gibco	Cat#11811-031
SuperSep Ace, 5-20%, 17well	WAKO	Cat#19415021
Amicon ultra-0.5 100KDA 96/PK	Millipore	Cat#UFC510096
D-MEM	Nissui	Cat#05915
Lipofectamine RNAiMAX	Invitrogen	Cat#13778150
Opti-MEM I Reduced Serum Medium	gibco	Cat#31985070
DMEM/F12	Gibco	Cat#11320082
GlutaMAX Supplement 100 mL	Gibco	Cat#35050061

Zenon Rabbit IgG Labeling Kit Alexa Fluor 488	Invitrogen	Cat#Z25302
Zenon Rabbit IgG Labeling Kit Alexa Fluor 555	Invitrogen	Cat#Z25305
Zenon Rabbit IgG Labeling Kit Alexa Fluor 647	Invitrogen	Cat#Z25308
D-MEM(High Glucose) with L-Glutamine, Phenol Red and Sodium Pyruvate	Fujifilm	Cat#041-30081
Zenon Mouse IgG1 Labeling Kit Alexa Fluor 647	Invitrogen	Cat#Z25008
Software and algorithms		
NIS-Elements ver 5.11	Nikon	RRID:SCR_014329
Gen5 ver 3.09	BioTek	RRID:SCR_017317
